# Measuring and modelling the response of *Klebsiella pneumoniae* KPC prey to *Bdellovibrio bacteriovorus* predation, in human serum and defined buffer

**DOI:** 10.1038/s41598-017-08060-4

**Published:** 2017-08-21

**Authors:** Michelle Baker, David Negus, Dhaarini Raghunathan, Paul Radford, Chris Moore, Gemma Clark, Mathew Diggle, Jess Tyson, Jamie Twycross, R. Elizabeth Sockett

**Affiliations:** 1 School of Life Sciences, University of Nottingham, Medical School, Queen’s Medical Centre, Nottingham, NG7 2UH UK; 2 School of Computer Science, Jubilee Campus, University of Nottingham, Wollaton Road, Nottingham, NG8 1BB UK; 3 Empath Pathology Services Reception Floor A, West Block, Queens Medical Centre, Nottingham University Hospitals NHS Trust, Nottingham, NG7 2UH UK

## Abstract

In worldwide conditions of increasingly antibiotic-resistant hospital infections, it is important to research alternative therapies. *Bdellovibrio bacteriovorus* bacteria naturally prey on Gram-negative pathogens, including antibiotic-resistant strains and so *B. bacteriovorus* have been proposed as “living antibiotics” to combat antimicrobially-resistant pathogens. Predator-prey interactions are complex and can be altered by environmental components. To be effective *B. bacteriovorus* predation needs to work in human body fluids such as serum where predation dynamics may differ to that studied in laboratory media. Here we combine mathematical modelling and lab experimentation to investigate the predation of an important carbapenem-resistant human pathogen, *Klebsiella pneumoniae*, by *B. bacteriovorus* in human serum versus buffer. We show experimentally that *B. bacteriovorus* is able to reduce prey numbers in each environment, on different timescales. Our mathematical model captures the underlying dynamics of the experimentation, including an initial predation-delay at the predator-prey-serum interface. Our research shows differences between predation in buffer and serum and highlights both the potential and limitations of *B. bacteriovorus* acting therapeutically against *K. pneumoniae* in serum, informing future research into the medicinal behaviours and dosing of this living antibacterial.

## Introduction

Infections caused by antibiotic-resistant Gram-negative bacteria have steadily risen over the past decade and pose a serious risk to public health. This is illustrated by the fact that Gram-negative bacteria are currently responsible for more than 30% of all hospital-acquired infections^1^ and are associated with high levels of morbidity and mortality in the Intensive Care Unit setting^2–5^. Consequently, new therapies effective against drug resistant isolates are urgently needed. One option mooted is the use of predatory bacteria, such as *Bdellovibrio bacteriovorus*, as therapeutic agents^6, 7^. *B. bacteriovorus* HD100 is a small (0.3 µm × 1 µm) Gram-negative bacterium that preys on other Gram-negative bacteria as part of its developmental lifecycle, which has a number of well-defined stages in liquid conditions^8^. During this lifecycle, the prey act as both food source and housing for the predator^9^. Firstly, highly motile attack phase predator- cells are propelled by a single flagellum until they encounter a prey cell, at which point they attach to the outer membrane and invade the prey periplasm, forming a predator+dead-prey structure called a bdelloplast. Once established within the periplasm, *B. bacteriovorus* begin filamentous replication by virtue of sequestration of prey cell nutrients. Depending on the prey and external environment, the typical lifecycle of *B. bacteriovorus* lasts 3–4 hours and when complete results in lysis of the prey and release of new predatory progeny. The number of predators released is dependent on the size of the prey cell^10^.

Understanding the dynamics of *B. bacteriovorus* predation under the *in vivo* conditions found in a treatment setting, is critical to developing this predatory bacterium as a “living antibiotic”. Formulating a dosage regime for any future administration of a living organism inside a complex system, such as the human body, requires a comprehensive understanding of the factors affecting predation. Previously published *in vitro* predation studies in buffer and bacteriological media have been informative^7, 11^ but need expanding towards physiologically relevant conditions. In order to address this issue, we employ an interdisciplinary approach to investigate experimentally and mathematically model the predation by *B. bacteriovorus* HD100 of an antibiotic-resistant, carbapenemase producing (KPC) clinical isolate of *Klebsiella pneumoniae* in whole human serum at 37 °C; conditions that better represent those found *in vivo. K. pneumoniae* KPC is an important human pathogen and member of the ESKAPE group^12^ of antibiotic resistant human pathogens with carbapenem resistance, which further limits the treatment options for patients. It is a major cause of nosocomial infections globally and has an associated high mortality rate^13^.

Early mathematical modelling of *B. bacteriovorus* predation, in buffers/simple lab media alone, includes the influential work of Varon and Zeigler^14^ who used a Lotka-Volterra model^15, 16^ to investigate predation on a *Photobacterium species*. Experimentally they used low concentrations of both predator and prey in a tri-salt buffer to determine the concentrations necessary for survival of *B. bacteriovorus* and to induce oscillatory waves of predatory behaviour in prey populations. They then used this data to estimate parameters for their Lotka-Volterra model, which predicted that “the prey density required to give *bdellovibrios* a 50% chance of survival was calculated to be at least 3.0 × 10^6^ cells per ml”, although this has been later disputed^17^. In contrast, driven by the potential therapeutic application, our model focuses on prey elimination rather than *B. bacteriovorus* survival. We take the Lotka-Volterra model as a basis for our model, but adapt it significantly to reflect the unique predation mechanism of *B. bacteriovorus*. Most significantly, our model allows us to capture temporal aspects of predation not accounted for in Varon and Zeiglers’ model, and also assess the impact of prey resistance and environmental variables on predation outcome.

Other early work includes a model of *B. bacteriovorus* predation on *Escherichia coli* which explicitly includes a bdelloplast stage with an incubation period during which the predator *B. bacteriovorus* grows and replicates inside prey^18^. In contrast to our model, this model only considered predation in buffer with no consideration of immune system effects, and only looked at short time scales (<10 hours after inoculation), allowing the authors to use a much simpler model than the one we present. We also note that the parameter estimates reported for progeny number (from *E. coli*) are larger than those that have since been observed experimentally. Later modelling work by Wilkinson^19^ and Hobley^20^ examined predation in the presence of a third ‘decoy’ species, either inert or live. These studies are an important step away from the previous tightly controlled two species model systems, and the presence of other bacterial species is important in assessing the clinical application of *B. bacteriovorus* against mixed bacterial infections. These studies, however, unlike the model presented in this work, consider predation in buffer rather than in a complex growth environment as would be the case in any future clinical application.

Most recently, Dattner^21^ modelled *B. bacteriovorus* predation in a habitat of sand suspended in buffer. The authors proposed an alternative approach to the standard (numerical integration based) parameter estimation approach used in analysis of non-linear dynamics. By taking a simplified ‘strategic’ model rather than a mechanistic, model the authors were able to use a statistical direct integral approach to estimate the model parameters and produced a fit to a small sample of experimental data points. Whilst this approach may give insights into predation dynamics in an artificially simplified experimental environment, it is unlikely to be suitable for the more complex environment that would be present in the clinical application of *B. bacteriovorus*, such as serum. In a whole human environment, several immunological and/or antimicrobial factors (such as antibodies, antimicrobial peptides and leukocytes) will act upon the predatory bacteria, potentially killing the predators before predation can occur. These factors are complex but important to understand and quantify as they will impact efficacy of *B. bacteriovorus* used as a therapeutic.

In this study, we used standard numerical integration to estimate the model parameters and simulate time course behaviour in human serum at the physiological temperature of 37 °C. This results in a complex parameterisation of the predation process, even for serum (albeit without the leukocytes that would be present in whole blood, an area that requires significant further experimentation). By capturing the mechanistic complexity of the experimental system, we are able to use our model to guide the design of experiments and to suggest mechanisms to further investigate. Furthermore, our model allows rapid prediction of predation outcomes over longer timescales, which would be difficult and labour-intensive to obtain experimentally.

Mathematical modelling of *B. bacteriovorus* predation cannot be thought of as a variation of more traditional animal predator-prey dynamics^22, 23^. The *B. bacteriovorus* intra-prey, life cycle length and uniformity leads to a synchronicity of prey bursting and replicated progeny-predator release, in controlled laboratory media (such as buffer), and when an almost equal number of predators to prey are present at the start of predation. Whilst this will not affect the long term (relative to life cycle length) dynamics of the model, it has a significant impact on the shorter term dynamics (less than the cycle length). Previous modelling work for predation in buffer^14^ did not include the impact that synchronous prey-bursting may have on the validity of the model at early time points, and the early mathematical models were not able to fully account for the differences between bacterial and animal predation. In this paper, we present a more clinically applicable ordinary differential equation (ODE) population-based model of bacterial predator-prey dynamics which is experimentally validated and informs upon both short- and long-term dynamics in buffer and human serum. We also consider some important aspects of predation that as yet have not been fully investigated. Firstly we consider the baseline conditions of predation in buffer, over longer time scales (up to 96 hours). This allows modelling of the prey outgrowing their predators, linking to reports from previous experimental work^14, 17^. Secondly, we mathematically model and experimentally determine the dynamics of *B. bacteriovorus* predation of *K. pneumoniae* in human serum; not the usual buffer employed for most previous predation studies. We quantify and demonstrate that pathogen predation in human serum does occur under certain conditions, that the dynamics of predation in serum is markedly different to that in buffer and that there is serum to serum variation.

A more complete understanding of the potential and limitations of predation in human serum is essential if ultimately *B. bacteriovorus* is to be considered for use as a living antibiotic. Both our experimental work and model show a pattern of delayed predation in serum initially lowering the *K. pneumoniae* load, but this is followed by prey regrowth, which will need to be fully understood in future work if we and other *Bdellovibrio* researchers are to prevent re-infection. The interdisciplinary approach we take here is crucial to achieving that aim, with the experimental work highlighting the marked differences between predation in serum and buffer, and the mathematical modelling providing insight into potential reasons for these differences. This paper takes important steps towards increasing our understanding of *B. bacteriovorus* predation in a realistic environment and provides a framework for further developing *B. bacteriovorus* as a potential therapeutic.

## Methods

### Bacterial strains, serum, media and growth conditions

*Klebsiella pneumoniae* EARSSU271 is a carbapenemase producing (KPC) clinical isolate and was provided via Dr Mathew Diggle (EMPATH Nottingham University Hospitals UK). *K. pneumoniae* was cultured on LB agar and YT broth at 37 °C. *B. bacteriovorus* HD100^24^ was cultured as described previously^25^. Briefly, *B. bacteriovorus* stocks were prepared by culture on stationary phase *E. coli* S17-1^26^ prey suspended in Ca/HEPES buffer (5.49 g/l HEPES free acid, 0.284 g/l calcium chloride dehydrate, pH 7.6). Predatory cultures were prepared by culturing of *E. coli* S17-1 in YT broth (5 g/l sodium chloride, 5 g/l peptone, 8 g/l tryptone, pH 7.5) at 37 °C with shaking for approximately 16 h. Stationary phase prey cells (3 ml) were then added to 1 ml of a previous *B. bacteriovorus* predatory culture in 50 ml Ca/HEPES buffer and incubated at 29 °C for at least 16 h until the culture had cleared. (Predator and prey cell numbers were enumerated from cultures as described in experimentation below). Human serum (pooled from male AB plasma) was sourced from Sigma (H4522, Sigma-Aldrich Corporation, St. Louis, MO, USA).

### Assaying reduction of *K. pneumoniae* numbers by human serum alone

*K. pneumoniae* was cultured in YT broth (20 ml) at 37 °C with shaking (200 rpm) for approximately 16 h. *K. pneumoniae* was pelleted by centrifugation (5,500 × g, 10 min, 29 °C) and resuspended to an optical density (OD_600 nm_) of 2.0 in whole human serum (Sigma H4522, Sigma-Aldrich Corporation, St. Louis, MO, USA). The suspension was serially diluted 1:10 with fresh human serum in a total volume of 3 ml. Serially diluted cultures were incubated at 37 °C with shaking (200 rpm) and cell numbers were enumerated periodically by the method of Miles and Misra^27^ on LB agar in triplicate with three biological repeats. This was done to assay for any cell number reduction by serum alone.

### Assaying reduction of *B. bacteriovorus* HD100 numbers by human serum alone

*B. bacteriovorus* predatory cultures (500 ml) prepared as described previously were passed through a 0.45 µm Millex filter (Millipore, Billerica, MA, USA) to remove any remaining starter-prey, *E. coli* S17-1. The filtered predatory culture was centrifuged (5,500 × g, 20 min, 29 °C) to pellet cells. The pellet was resuspended in 500 µl Ca/HEPES to give a 1000 × concentrated suspension typically containing approximately 4 × 10^10^ pfu/ml of *B. bacteriovorus*. The suspension was serially diluted 1:10 with Ca/HEPES buffer in a total volume of 500 µl. Serially diluted *B. bacteriovorus* suspension (450 µl) was added to 4.45 ml of whole human serum and suspensions were incubated at 37 °C with shaking (200 rpm). Cell numbers were determined periodically by serial dilution and enumeration of *B. bacteriovorus* plaques in a soft YPSC overlay agar (0.25 g/l magnesium sulphate, 0.5 g/l sodium acetate, 1.0 g/l Bacto peptone, 1.0 g/l yeast extract, pH 7.6; adjust to 0.25 g/l anhydrous calcium chloride after autoclaving). For YPSC bottom agar, add 10 g/l agar. For YPSC overlay agar, add 6 g/l agar) containing a lawn of *E. coli* S17-1 as described previously^25^. Enumerations were performed in duplicate (two technical repeats) with two biological repeats.

### Predation assays in buffer and serum

Predatory cultures of *B. bacteriovorus* (50 ml) prepared as described earlier in methods were passed through a 0.45 µm Millex filter to remove any remaining *E. coli* S17-1 and the filtered predatory culture was centrifuged at 5,500 × *g*, 20 min, 29 °C. The pellet was resuspended in 1 ml of Ca/HEPES to give a 50 × concentrated preparation typically containing approximately 5 × 10^9^ cfu/ml of predatory *B. bacteriovorus*. *K. pneumoniae* prey was cultured in YT broth (20 ml) at 37 °C with shaking (200 rpm) for approximately 16 h. Prey cells were pelleted by centrifugation (5,500 × g, 10 min, 29 °C) and resuspended to an optical density (OD _600 nm_) of 1.0 (equivalent to approximately 5 × 10^9^ cfu/ml) in 5 ml whole human serum (Sigma H4522, Sigma-Aldrich Corporation, St. Louis, MO, USA) or 5 ml Ca/HEPES buffer. Predation assays were started by combining 5 ml of prey suspension with 500 µl of concentrated *B. bacteriovorus* suspension or 500 µl of Ca/HEPES as a control in a sterile Falcon tube (50 ml) and incubated at 37 °C in a rotary shaker (200 rpm). Starting predator and prey numbers were determined by enumeration of both prey and predator populations. Changes in prey population were enumerated by method of Miles and Misra plating in triplicate. Changes in predator population were determined by serial dilution and enumeration of *B. bacteriovorus* plaques in a soft YPSC overlay agar.

### Fluorescence microscopy

For fluorescence microscopy, predation assays in serum or buffer were prepared as described in methods. To be able to visualise small predators in the visually challenging serum environment, we used *B. bacteriovorus* HD100 expressing, constitutively from the chromosome, Bd0064mCherry fluorescent protein (named HD100 mCherry) described in Willis *et al*.^28^. Aliquots (10 µl) of predation assay were withdrawn for microscopy and placed on a solid 1% agarose pad prepared in Ca/HEPES buffer. Fluorescent microscopic images were acquired using a Nikon Ti-E Eclipse inverted microscope (excitation 555 nm / emission 640 nm, 1 s exposure).

### Analysis of *B. bacteriovorus* morphology in serum

MicrobeJ image analysis software^29^ was used to detect fluorescent *B. bacteriovorus* HD100 mCherry cells and measure the roundness (*4* × *area / (π* × *major axis* ^2^) of each individual cell. A roundness cut off of 0.65 was selected to distinguish between cells with typical *B. bacteriovorus* vibroid morphology (roundness score < 0.65) and those with an abnormal round morphology (roundness score ≥ 0.65). Manual inspection of cells near the cutoff validated that the cells were correctly called.

### Analysis of *K. pneumoniae* cell area in serum

MicrobeJ image analysis software was used to detect *K. pneumoniae* prey cells in serum and measure the area of each individual cell. The mean cell area of the *K. pneumoniae* KPC population in serum was determined at 0 hours (n = 271 individual measurements) and 96 hours (n = 170 individual measurements).

### Pre-exposure of predator and prey to serum followed by predation in buffer

To test whether delay effects seen in predation in serum were due predominately to serum-induced changes in predator or prey, additional experiments were performed. *B. bacteriovorus* predatory cultures (50 ml, typically containing approximately 1 × 10^8^ pfu/ml) prepared as described were passed through a 0.45 µm Millex filter to remove any remaining *E. coli* S17-1 and the filtered predatory culture was centrifuged at 5,500 × *g*, 20 min, 29 °C. The pellet was resuspended in 5 ml of whole human serum and incubated at 37 °C with shaking (200 rpm) for 2 h. *K. pneumoniae* prey was cultured in YT broth (20 ml) at 37 °C with shaking (200 rpm) for approximately 16 h. Prey cells were pelleted by centrifugation (5,500 × g, 10 min, 29 °C) and resuspended to an optical density (OD _600 nm_) of 1.0 (equivalent to approximately 5 × 10^9^ cfu/ml) in 5 ml whole human serum and incubated at 37 °C with shaking (200 rpm) for 2 h. Following exposure to serum, predator and prey suspensions were pelleted by centrifugation (5,500 × g, 10 min, 29 °C). *K. pneumoniae* pellets were resuspended in 5 ml of Ca/HEPES and *B. bacteriovorus* HD100 pellets were resuspended in 500 µl of Ca/HEPES. To begin predation assays, 500 µl of *B. bacteriovorus* suspension was added to 5 ml of *K. pneumoniae* KPC suspension in Ca/HEPES and incubated at 37 °C with shaking (200 rpm). Predation was determined by enumeration of both prey and predator populations as described. Experiments were performed with three biological repeats. Prey enumerations were performed with technical triplicates and predator enumerations performed with technical duplicates.

### Measure of complement activity during predation

To establish how complement levels changed during predation in serum, haemolysis assays were carried out using standard protocols as described earlier with minor modifications^30, 31^. Predation assays with *B. bacteriovorus* (approximate starting concentration 1 × 10^8^ cfu/ml) and *K. pneumoniae* (approximate starting concentration 5 × 10^9^ cfu/ml) in whole human serum were prepared as described previously and incubated at 37 °C with shaking (200 rpm). Aliquots of serum (1 ml) were removed at each time point and passed through a 0.2 µM Millex syringe driven filter to remove both predator and prey. Aliquots of filtered serum were immediately frozen (−20 °C) prior to determining remaining complement activity. The classical complement pathway activity was tested with sheep erythrocytes opsonised with Rabbit anti-sheep red blood cell stroma IgG antibodies (Sigma-Aldrich). Sheep erythrocytes were washed twice in 0.9% NaCl and twice in GVB^++^ (3.12 mM barbitone, 0.97 mM sodium barbiturate, 145 mM NaCl, 0.83 mM MgCl_2_, 0.25 mM CaCl_2_ with 0.1% gelatin). The erythrocytes were resuspended in GVB^++^ to 10^9^ cells/ml and incubated with the antibody for 30 minutes at 37 °C, followed by incubation on ice for 45 minutes. The opsonized sheep cells (EA) were washed twice in GVB^++^ and resuspended to 10^8^ cells/ml. The serum samples were serially diluted 2-fold in 50 µl of GVB^++^ and incubated with 25 µl of EA at 37 °C for 30 minutes. The assay was stopped by adding 125 µl of cold GVB^++^ buffer. The assay controls included 100% lysis control (25 μl of EA lysed in 175 of μl deionized water) and blank (25 μl of EA incubated in 175 μl of GVB^++^). The samples were centrifuged briefly (5000 x *g*, 2 minutes) to pellet intact erythrocytes and the haemolysis was measured spectrophotometrically as the amount of released haemoglobin (OD at 415 nm) in a microplate reader.

### Determination of *B. bacteriovorus* HD100 burst size

The average number of predator progeny released from *K. pneumoniae* prey cells following infection and replication was determined by video microscopy to aid parameterization. Briefly, *K. pneumoniae* cells were prepared and infected with *B. bacteriovorus* HD100 following the protocol described for predation assays with *K. pneumoniae* cells resuspended in Ca/HEPES buffer. Predation assays were incubated at 37 °C with shaking (200 rpm) for 2 h and 10 µl of the suspension was then removed from the assay and placed on a solid 1% agarose pad surface prepared in Ca/HEPES buffer. Microscopic images were acquired over several hours at room temperature every 150 s as described previously^32^. Microscopic images were encoded into time-lapse movies and the number of progeny released from individual prey cells counted to give the average burst size (Supplementary material, Figure S1).

### Mathematical Modelling

**Figure 1:**
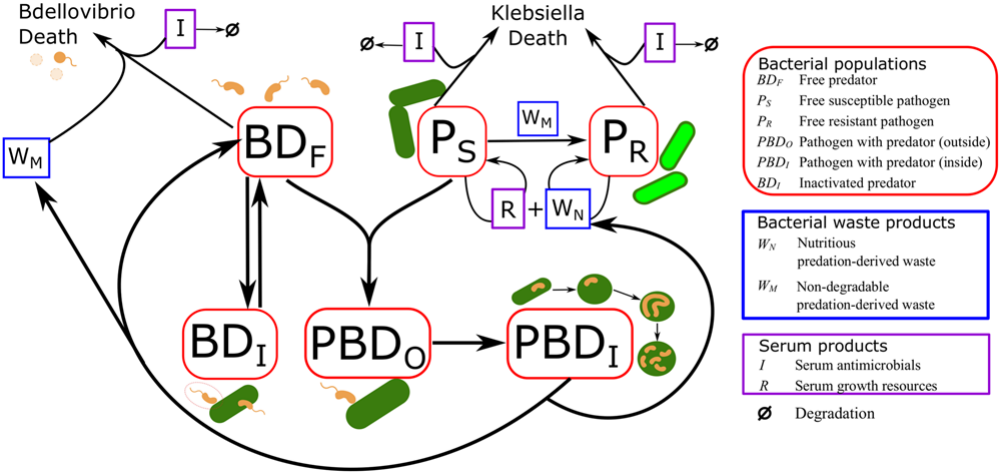
Schematic diagram of relationship between model variables based on biological mechanisms. The Ca/HEPES buffer model has eight variables: free *B. bacteriovorus* predators (*BD*_*F*_), uninfected predation-susceptible *K. pneumoniae* prey (*P*_*S*_), uninfected predation-resistant *K. pneumoniae* prey (*P*_*R*_), prey with predator attached outside (*PBD*_*O*_), invaded prey with predator on the inside (*PBD*_*I*_), inactivated predator (*BD*_*I*_), nutritious waste (*W*_*N*_) and non-degradable waste (*W*_*M*_). The serum model has an additional two variables serum growth resources (R) and serum antimicrobials (I). Detailed description of the biological mechanisms can be found in the supplementary materials.

We developed mathematical models of *K. pneumoniae* KPC predation by *B. bacteriovorus* HD100 in both buffer and human serum. The motility of the bacteria and incubation in a rotary shaker led to a relatively homogenous environment, hence we used population based ODEs. The models we developed consider how the populations of predator and prey bacteria change over time in different environments. The models were influenced by previous modelling of *B. bacteriovorus* predation^14, 18–20^ as well as a model of the interaction of bacteria with serum^33^. The serum model presented here has ten variables (Table 1) and 27 parameters (Table 2). We use a reduced version of the serum model to simulate predation in buffer, discounting the serum specific variables and related parameters. The full model is shown diagrammatically in Fig. 1, with full details of the model given in the supplementary materials. Experimental results were used to set initial conditions for the model, estimate rate parameters for the mechanisms of predation, and validate the model for serum and for buffer (see supplementary materials for a detailed description). All model simulation, parameter fitting and analysis were performed in MATLAB^34^. Combinations of the model variables were used as reporting variables to emulate experimental prey and predator enumeration. Individual model variables were also analysed to explore predation dynamics that cannot be observed experimentally.

**Table 1:** Model variables and definitions for both the buffer and human serum predation models.

Variable	Definition
*BD*_*F*_	Free predator
*P*_*S*_	Free susceptible pathogen
*P*_*R*_	Free resistant pathogen
*PBD*_*O*_	Pathogen with predator (outside)
*PBD*_*I*_	Pathogen with predator (inside)
*BD*_*I*_	Inactivated predator
*W*_*N*_	Nutritious predation-derived waste
*W*_*M*_	Non-degradable predation-derived waste
*I*	Serum antimicrobials*
*R*	Serum growth resources*

The variables marked with *are present in the serum model only.

**Table 2:** Parameters used in both buffer and serum models with definitions and units.

Parameter	Definition	Units
*k*_1_^*early*^	Early predator-pathogen binding rate	ml cell^−1^ s^−1^
*k*_1_^*late*^	Late predator-pathogen binding rate	ml cell^−1^ s^−1^
*k*_2_	Predator internalization rate	s^−1^
*k*_3_	Predator reproduction rate (time from entry to exit)	s^−1^
*k*_4_	Transient predator-pathogen binding rate	s^−1^
*V*_*max*_	Maximum pathogen growth rate on serum	s^−1^
${V}_{{\max }}^{w}$	Maximum pathogen growth rate on predation-derived waste	s^−1^
*E*	Efficiency of serum resource metabolism	arb. units
*E*_*w*_	Efficiency of predation-derived waste resource metabolism	arb. units
*s*	Serum growth resource at half maximal growth	arb. units
*s*_*w*_	Waste growth resource at half maximal growth	arb. units
*d*_*P*_	Rate of accumulation of predation-derived waste	s^−1^
*d*_*BD*_	Debris produced from predator cell death	s^−1^
*d*_*I*_	Natural antimicrobial inactivation rate	s^−1^
${d}_{{\max }}^{{BD}}$	Maximal predator removal rate due to dead predator debris	s^−1^
${d}_{{\min }}^{{BD}}$	Baseline predator removal rate due to dead predator debris	s^−1^
*h*	Rate of change of predator removal rate	arb. units
*a*_*BD*_	Predator removal rate by antimicrobials	s^−1^
*a*_*P*_	Prey removal rate by antimicrobials	s^−1^
*c*_*BD*_	Predator dependant antimicrobial inactivation rate	s^−1^
*c*_*P*_	Pathogen dependant antimicrobial inactivation rate	s^−1^
*f*	Rate of prey transition to resistance	s^−1^
*n*	Fractional index of pathogen-antimicrobial interaction	dimensionless
*ρ*	Predator progeny number	dimensionless
*λ*	Natural predator death rate	s^−1^
*ν*	Proportion of viable invaded prey	dimensionless
*δ*	Reduced attachment length at early timepoints	h^−1^
*I*_0_	Initial serum antimicrobial concentration	arb. units
*R*_0_	Initial serum growth resource concentration	arb. units

## Results

### Predation of *K. pneumoniae* KPC in defined buffer

**Figure 2:**
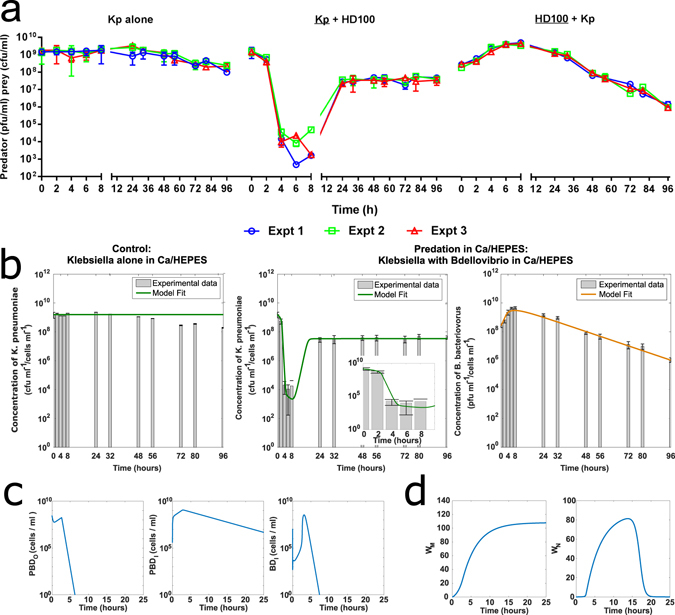
Predation Experimentally Measured and Modelled in Ca/HEPES buffer. (**a**) Graphs show individual biological repeats- separate experiments (Expt) of the live bacterial counts from predation in buffer experiments, results from each individual biological experiment are represented by the same colour line on each graph. Changes in the population of both *K. pneumoniae* prey (Kp + HD100) and *B. bacteriovorus* HD100 predator (HD100 + Kp) were enumerated periodically during the predation assay. As a control, prey suspended in Ca/HEPES was enumerated periodically in the absence of *B. bacteriovorus* HD100 predator (Kp alone). (**b**) Prey concentration (green line) initially shows a 6 log_10_ reduction by 6 hours (see inset) before regrowth overtakes predation effects. Predator concentration (orange line) grows in line with the prey death for the first six hours then reduces, in line with experimental results. Experimental results reproduced from A shown as a bar plot, the model simulation lies within the error bars of the experimental results. (**c**) Model simulation results showing prey with a predator attached (PBD_O_), prey with a predator inside (PBD_I_) and inactivated predators (BD_I_) over time. Attachment drops off after 5 hours due to prey becoming resistant.(**d**) Model simulation of waste accumulation over time. Non-degradable waste increases monotonically leading to prey resistance whilst nutritious waste initially increases but is then depleted as it is used by the growing predominently resistant prey. Parameter values and further model validation are detailed in supplementary material. The model gives a good fit to the experimental data.

Experimentally, predation of *K. pneumoniae* KPC in a buffer environment at 37 °C was found to be highly reproducible (Fig. 2a) and resembled predation results reported for other Gram-negative bacteria such as *E. coli, Acinetobacter* spp. and *Shigella* spp. in buffer environments^7, 17^. Figure 2a shows a rapid reduction in the viability of *K. pneumoniae* in the presence of *B. bacteriovorus* HD100 with an approximate four log reduction of prey numbers by four hours. Reduction of prey was concomitant with an increase in predator numbers which is indicative of predation. These early time point results reflect the typical predatory lifescycle reported for *B. bacteriovorus* in lab strains *of E. coli*: an initial period of attachment and invasion is followed by prey death and filamentous replication of predator within a bdelloplast and lysis of the prey after 3–4 hours^6^. The timing and magnitude of the reduction in prey viability suggest that these initial predation events occurred, in buffer, with a high level of synchronicity which was further confirmed by additional microscopy (Supplementary Figure S2).

The mathematical model of predation of *K. pneumoniae* KPC in buffer by *B. bacteriovorus* HD100 gave a good fit to the experimental data (Fig. 2b). The model includes the effect of debris from predation (termed “predation-derived waste” model) on predators and prey. We consider that this waste will have two main effects, firstly some can be used by the prey for metabolism (W_N_), leading to growth of the prey cell population and secondly, any unused debris will remain in the media (W_M_) and contribute to the resistant phenotype suggested by the experimental data. The model followed the same trends seen experimentally, with an initial drop in prey numbers followed by a rapid regrowth within 24 hours. Such rapid regrowth is not reported for laboratory *E. coli* strains in buffer and may indicate proteolysis of predator by prey, liberating amino-acids for prey nutrition. The parameter estimation predicts an internalisation time of 11.5 minutes (*k*_2_ in mins) and a replicated predator exit time of 4 hours (*k*_3_ in hours) in *K. pneumoniae*, which are broadly in line with published times for *E. coli* ^35^, although species and strain specific variation is to be expected. In addition to predator and prey counts which can be measured experimentally, the model allows the examination of other quantities, such as bdelloplast numbers and total serum antimicrobial activity, which are difficult to measure quantitatively *in vitro*, allowing the underlying dynamics to be investigated further. The model, with best-fit parameters, predicts that in buffer, *B. bacteriovorus* concentration increases at early time points (up to approximately 8 hours), then declines slowly over time at a rate that matches the experimental data and is in line with previous studies^14^. Figure 2c shows predators attached to prey (PBD_O_), prey with predators inside (PBD_I_) and transiently attached predators (BD_I_) over time and declining PBD_I_ and PBD_O_ concentrations show that new predation is halted after the first 5 hours, in buffer, indicating that all the *K. pneumoniae* prey bacteria that have not been invaded by a predator are now resistant in the buffer model. The results from both the model and experimental work follow a very similar trend to experimental results for in-buffer predation of a different bacterium *Erwina caratovora ssp. caratovora* presented in Shemesh and Jurkevitch^17^, which show the same pattern of a large drop in prey numbers due to predation followed by an apparently resistant regrowth. Our model here supports their conclusions that plastic resistance induced by means of environmental changes could explain the experimental results and suggests that the amount of resistance is dependent upon the build-up of debris from successful predation.

**Figure 3:**
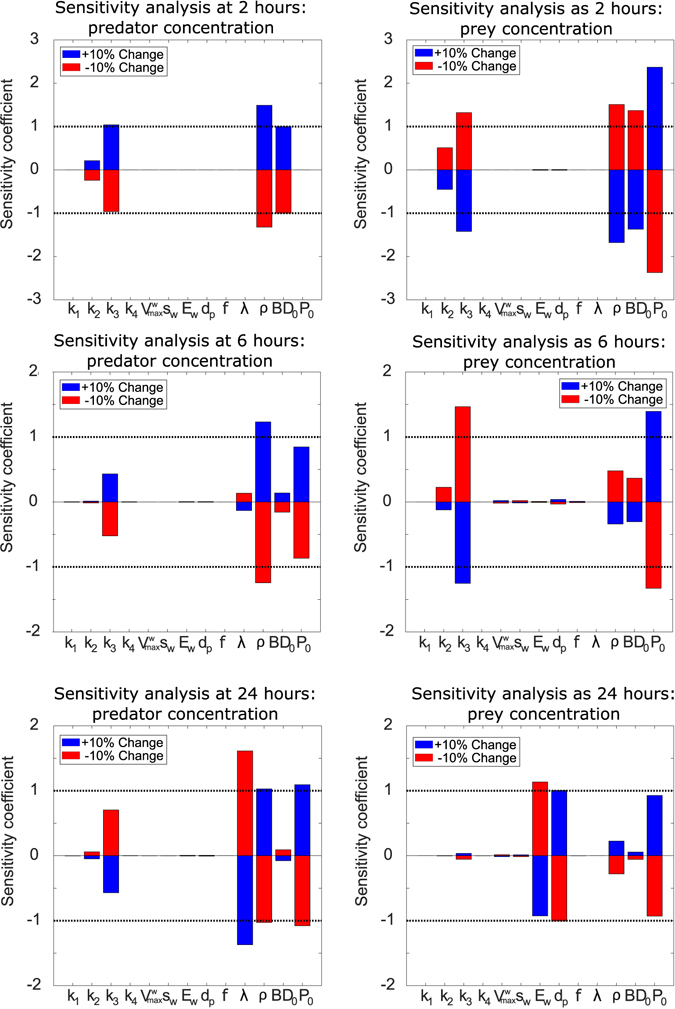
One-at-a-time sensitivity analysis of a ± 10% change in each parameter at three different time points. The variation in parameter sensitivity at different times reflects the different mechanisms dominating predator and prey interactions at those times. The sensitivity coefficient is scaled such that a value of ± 1 represents a ± 10% change in concentration as a result of a ± 10% change in parameter value. Parameter values detailed in Supplementary Table S2.

To test the robustness of the model we conducted a sensitivity analysis at several key time points representing different stages of the simulation for the buffer model. Figure 3 shows a one-at-a-time sensitivity analysis for a 10% change in parameter values at 2, 6 and 24 hours, calculated as described in the supplemental material. This showed that the model is largely robust to 10% variations in parameter values. In the short term, the model is sensitive to small changes in ρ, k_3_ and P_0_, but the longer term dynamics are not affected. Variation in the natural death rate of predators, λ, has a long term effect on the *B. bacteriovorus* concentration. It should also be noted that while the system is sensitive to these parameters, they are not all directly amenable to manipulation in an experimental setting.

In the first 6 hours of the simulation, *K. pneumoniae* concentration in the buffer model is sensitive to predation related parameters (*ρ*, *k*_3_ and starting concentrations) but in the longer term, the model is not sensitive to these parameters. This can be explained by longer-term prey regrowth, shown experimentally in Fig. 2a. The 10% variation in these parameters changes the temporal dynamics of predation but the total amount of predation remains constant. We also looked at both large and smaller parameter changes and found the results to be the same (results not shown). Longer term (24 hours), the *K. pneumoniae* concentration is sensitive only to changes in the efficiency of metabolism of predation-derived waste (*E*_*W*_). This clearly demonstrates that in the closed experimental system it is the regrowth of *K. pneumoniae* that dominates experimental outcome. This occurs as a result of changing conditions over the course of the experiment, often neglected in previous work. Whilst the amount of prey-derived waste present (but not the efficiency of its usage) could possibly be manipulated experimentally *in vitro*, it cannot be altered *in vivo*, the host-relevant state, so we did not consider that further.

The sensitivity analysis highlights an important concept. Whilst we, and others previously, report on predation and factors affecting the amount and rate of predation, the closed batch experimental model may give predation dynamics that differ to what might be seen in a medically relevant circulation- perfused, physiological situation. However, no such quantitative perfused studies exist *in vivo* yet, as they are very experimentally challenging. Considering the dynamics of serum-based predation in batch is a crucial stepping stone to such future work.

### Reduction of predator cell numbers in whole human serum alone

**Figure 4:**
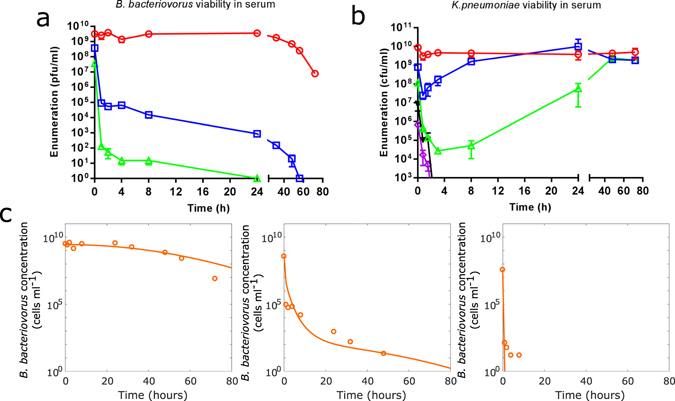
(**a**) Reduction of *B. bacteriovorus* HD100 by whole human serum. A serially diluted preparation of *B. bacteriovorus* HD100 was inoculated into whole human serum and viability measured over a 72 h period. (**b**) Reduction of *K. pneumoniae* concentration by whole human serum. A serially diluted preparation of *K. pneumoniae* was inoculated into whole human serum and viability measured over a 72 h period by Miles and Misra. (**c**) Model simulations of *B. bacteriovorus* reduction in human serum for three different initial conditions. Solid line show simulation result whilst the circles show the experimental results (replicated from panel a). Parameter values are given in Supplementary Table S2, in the supplementary materials.

To better understand the effect of human serum on the reduction of *B. bacteriovorus* HD100 and *K. pneumoniae* and to define parameters for our mathematical model, we separately determined the viability of *B. bacteriovorus* HD100 and *K. pneumoniae* in a serum environment over a range of starting concentrations. Reduction of *B. bacteriovorus* in human serum was strongly dependent on the initial bacterial concentration (Fig. 4a). An initial *B. bacteriovorus* concentration of 3.67 × 10^7^ pfu/ml was found to be cleared from human serum within 24 h. In contrast, *B. bacteriovorus* was found to persist up to 72 h in serum when present at a 100-fold higher initial concentration of 3.17 × 10^9^ pfu/ml. To further investigate these dynamics, we developed a model of *B. bacteriovorus* survival. In order to obtain the concentration-dependent behaviour seen in the experimental results we made the *B. bacteriovorus* degradation rate proportional to time, serum antimicrobials and increasing dead *B. bacteriovorus* cell debris in the serum (This is not needed in the buffer model where *B. bacteriovoru*s is not lysed by serum antimicrobials). The serum model gives a very early (1 hour) fall in *B. bacteriovorus* concentration over time that follows the same trends as the experimental data (Fig. 4a) for all starting concentrations we considered (Fig. 4c). In the model, initial removal of *B. bacteriovorus* is due to serum antimicrobials, and the model predicts that these are exhausted within the first hour of the simulation in the closed batch. However, reduction in *B. bacteriovorus* continues experimentally, (Fig. 4a), past 1 hour, over and above the natural death rate (λ). Removal of *B. bacteriovorus* at these times, in the model, is due to the effect of deleterious products released into the serum from the damaged predators. We hypothesise these products may include hydrolytic predatory enzymes detached from their usual internal predator self-protection proteins^36^. We also considered a simpler model for *B. bacteriovorus* reduction, with no antimicrobial-independent degradation. Whilst this gave a good fit to experimental data at a low initial concentration (3.67 × 10^7^ pfu/ml), it was not able to account for *B. bacteriovorus* reduction at higher initial concentrations ( > 3.67 × 10^7^ pfu/ml).

### Reduction of prey cell numbers in whole human serum alone

**Figure 5:**
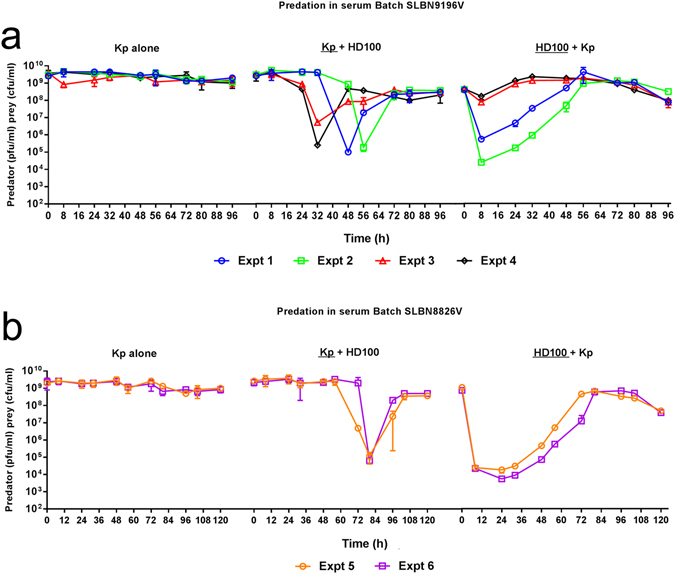
Predation in two different batches of whole human serum. **(a)** Serum batch SLBN9196V. **(b)** Serum batch SLBN8826V. Graphs show individual biological repeats of the live bacterial counts from predation in serum experiments, results from each individual biological experiment (Expt) are represented by the same colour line on each graph. Changes in the population of both *K. pneumoniae* prey (Kp + HD100) and *B. bacteriovorus* HD100 predator (HD100 + Kp) were enumerated periodically during the predation assay. As a control, prey suspended in serum was enumerated periodically in the absence of *B. bacteriovorus* HD100 predator (Kp alone). Despite illustrating some differences, similar patterns were seen across all 6 experimental repeats.

The survival of *K. pneumoniae* prey in human serum was similarly investigated over a range of starting concentrations (~5 × 10^5^ cfu/ml to ~9 × 10^9^ cfu/ml). Again, removal of bacteria from human serum was found to be strongly dependent on the initial concentration of bacterial cells present (Fig. 4b). Despite initial drops in viability, starting concentrations of *K. pneumoniae* cells above 7.76 × 10^6^ cfu/ml were found to recover and persist in serum following a period of regrowth. In contrast, lower starting concentrations swiftly dropped below our limit of detection (<1 × 10^3^ cfu/ml) and did not recover by 72 h. We used all the values for experimentally-determined reduction of *K. pneumoniae* in serum (Fig. 5a) to fit the *K. pneumoniae* growth parameters in our serum model. We assumed an initial condition for serum antimicrobials (*I*) from the best-fit parameters we obtained from parameter fitting the *B. bacteriovorus* reduction data. This modelling and experimentation guided us in selecting appropriate predator to prey starting concentrations for predation assays in human serum, reported below.

### Predation of *K. pneumoniae* (KPC) in human serum

Having measured and modelled the interactions between serum prey and predators alone, and having quantified the dynamic responses that alter their phenotypes in response to the serum surroundings; we then compared predation in serum versus that in buffer.

To investigate and model potentially important differences between predation in buffer and biological fluid, we repeated our buffer based assays in batches of human serum where factors other than predator-prey interaction could ameliorate bacterial numbers. Selection of initial predator to prey ratio for predation experiments in human serum was informed by the viability of *K. pneumoniae* KPC and *B. bacteriovorus* in serum reduction assays reported herein (Fig. 4a and b), and were chosen to be high enough that *K. pneumoniae* would not be cleared by serum alone, as in an established infection. As no accurate OD_600__nm_ can be measured for small *B. bacteriovorus* cells, accurate predator concentrations cannot be determined at the time of inoculation. Therefore, an approximate but tight range of initial predator concentrations were achieved by establishing a highly reproducible protocol. Accurate initial predator numbers for such experiments are enumerated by post-experiment viable counting with results available after the experiment (these are plotted in Fig. 5 and subsequently used in the mathematical modelling).

An initial concentration of approximately 5 × 10^9^ cfu/ml was selected for *K. pneumoniae* KPC prey on the basis that initial concentrations of this size were found to persist in serum for the duration of the assay (96 h) with only a minimal, early loss of viability (Fig. 4b). Therefore, significant reductions in prey viability, compared to controls, could be attributed to *B. bacteriovorus*, rather than solely any antimicrobial effect of the serum environment. Conversely, an initial concentration of approximately 5 × 10^8^ pfu/ml was chosen for *B. bacteriovorus* as starting concentrations of this size were found to steadily decrease in viability, in serum alone, with complete removal of predators occurring by 58 hours. This natural serum-related decrease in predators in the absence of prey allows us to attribute any rise in *B. bacteriovorus*, coupled with a decrease in *K. pneumoniae*, as an indicator of true predation causing predator replication. In addition the level of *B. bacteriovorus* used is similar to a topical dose applied to optical membranes of rabbit eyes, and reported as safe by Romanowski *et al*.^37^.

In contrast to assays performed in buffer, the dynamics of predation in serum were found to be more variable and the synchronicity observed in buffer was lost. However, some common trends did emerge. Predation and reduction of *K. pneumoniae* was observed in all six independent experiments using two different batches of human serum, (Fig. 5a and b) as determined by a decrease in prey population coupled with an increase in predator numbers, but predation was invariably followed by regrowth of the prey population. However, the length of time before measurable predation occurred and the extent to which it occurred, was found to be variable between the independent experiments, despite the small range of initial predator and prey concentrations, which ranged between 4.15 × 10^8^−1.2 × 10^9^ pfu/ml and 1.5–3.33 × 10^9^ cfu/ml respectively. The length of delay before a measurable reduction in the prey population was seen correlated with the *B. bacteriovorus* viable count measured at eight hours. For example, in experiments 3 and 4, in which the least 8hr predator death occurred, (<1 Log_10_ reduction in predator concentration at eight hours), we see the earliest predation-effected decrease in prey concentration (between 24 and 32 hours). Conversely, in experiments 5 and 6, in which the most 8hr predator death occurs (>4 Log_10_ reduction in predator concentration at eight hours) we see the latest decrease in prey concentration (between 72 and 84 hours). This delay in predation, seen in all the independent experiments, is very different to the predation kinetics in buffer and important to appreciate when considering possible therapeutic applications.

**Figure 6:**
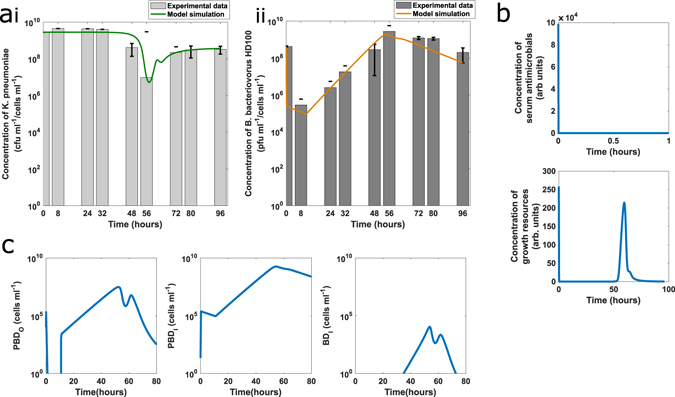
Model simulations of predation in human serum in comparison to experimental data, validating parameters estimated. **(a)** Model simulations with the best fit parameters (given in Supplementary Table S2) give a good fit to experimental data (mean of experimental repeats 1 and 2 from Fig. 5), with comparable dynamics. The drop in prey concentration due to predation and corresponding rise in predator numbers seen in the experimental data is replicated in the numerical simulation (**b)** The predators attached to prey (PBD_O_), predators inside prey i.e. bdelloplast (PBD_I_) and inactivated predators (BD_I_) are shown plotted against time. All three plots show a halting of predation for the first approximately 15 hours, then a continuing of the predatory cycle. By around 56 hours all the prey have become resistant. **(c)** The simulated serum antimicrobial concentrations showed that the antimicrobial activity of the serum is used up quickly (within the first 10 minutes of the simulation). Initial serum growth resources in the simulation are also quickly depleted, but an increase in growth resources is seen after 50 hours as lysis of bdelloplasts starts to release nutrients into the system.

**Figure 7:**
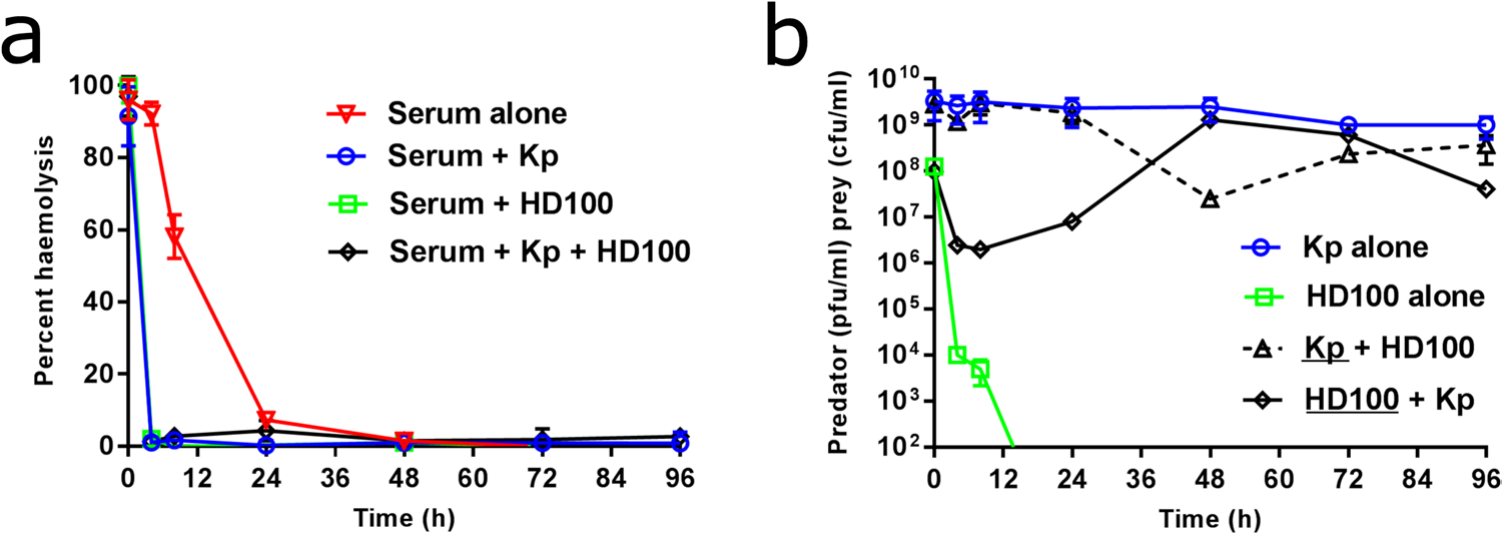
(**a**) Classical pathway complement activity of human serum incubated in presence of predator and/or prey as measured by percent haemolysis of sensitised erythrocytes. Human serum was incubated at 37 °C alone as a control (Serum alone) or in the presence of *K. pneumoniae* (Serum + Kp), in the presence of *B. bacteriovorus* HD100 (Serum + HD100) or in the presence of both *K. pneumoniae* and *B. bacteriovorus* HD100 (Serum + Kp + HD100). Serum was periodically sampled, filtered (0.2 µM) and diluted to 25% prior to measuring serum complement activity as determined by percent haemolysis of erythrocytes. CH_50_ serum values were calculated from these data and these are included in supplementary materials, Supplementary Table S1. (**b**) Enumeration of live bacterial populations incubated in human serum used for determining classical pathway complement activity. Predator and prey numbers in human serum were determined immediately prior to filtering (0.2 µM) and filtered aliquots were subsequently used for determining complement activity (panel a).

The mathematical model (Fig. 6a) suggests that the delay in predation we observe in serum cannot be explained solely by the initial death of *B. bacteriovorus*, as predation then proceeds at a much slower rate than that measured and modelled in buffer. Nor can this decreased rate of predation be due to continuing clearance of the predator population by the antimicrobial component of the serum, as the model predicts serum antimicrobials are exhausted within a few minutes of the start of the predation experiments (Fig. 6b). This has been confirmed experimentally, (Fig. 7a) and is discussed in detail later. Instead, the mathematical model suggests that a period of reduced attachment of predators to prey is also required to give the dynamics observed. Therefore, to better understand the dynamics of predation in serum and to gain insights into potential causes for this delay, we performed a microscopic study of predation in serum.

**Figure 8:**
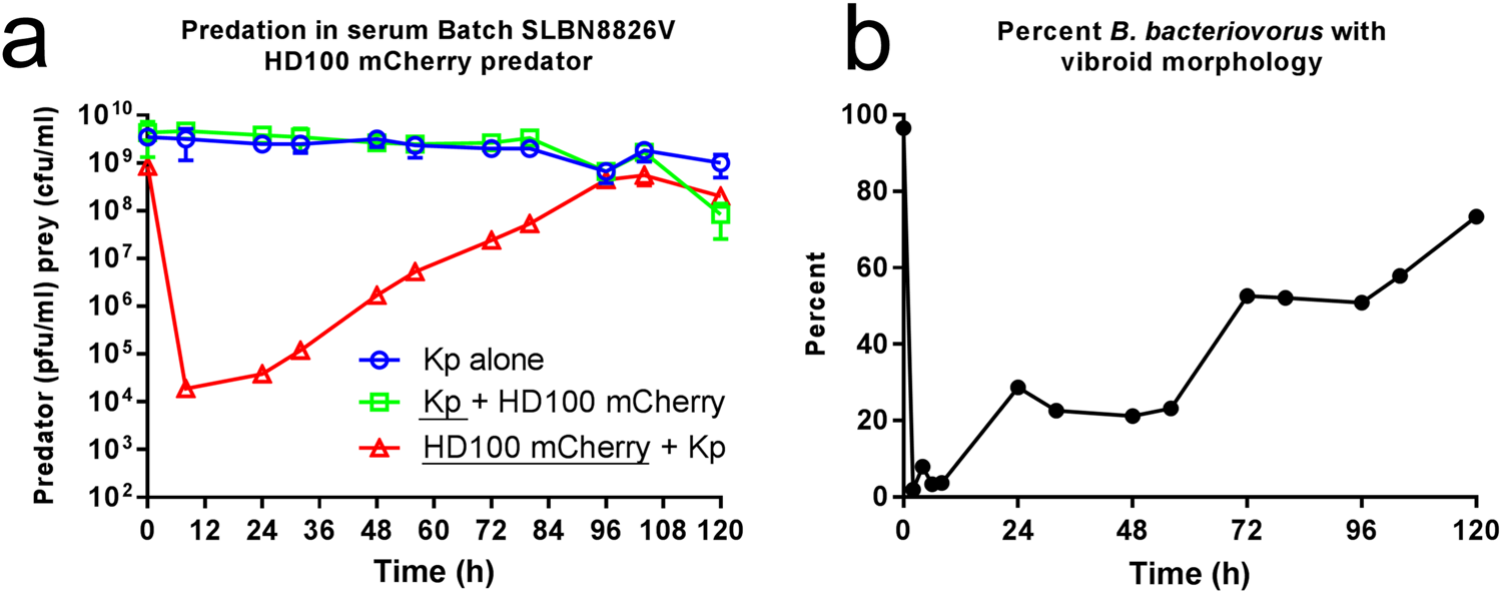
(**a**) Predation in whole human serum by HD100 mCherry fluorescent predator. Changes in the population of both *K. pneumoniae* prey (Kp + HD100 mCherry) and *B. bacteriovorus* HD100 mCherry predator (HD100 mCherry + Kp) were enumerated periodically during the predation assay. As a control, *K. pneumoniae* prey suspended in serum was enumerated periodically in the absence of *B. bacteriovorus* HD100 mCherry predator (Kp alone). **(b)** Microscopic analysis of *B. bacteriovorus* morphology in human serum with *K. pneumoniae* prey. MicrobeJ software was used to automatically detect mCherry tagged fluorescent *B. bacteriovorus* cells and measure the roundness (*4 × area / (π × major axis* ^2^) of each individual cell at each time point. A roundness cut off of 0.65 was selected to distinguish between cells with typical vibroid morphology (roundness score < 0.65) and those with an abnormal round morphology (roundness score ≥ 0.65). Number of cells analysed ranged between 1026 and 109 cells. Exact sample sizes for each time point are given in Supplementary material, Figure S4.

We used *B. bacteriovorus* HD100 expressing fluorescent mCherry protein (HD100 mCherry) in order to visualize small predator cells microscopically in the visually complex medium of serum. Predation of *K. pneumoniae* prey by HD100 mCherry was verified (as this is genetically a different predator to HD100) in Fig. 8a. These results again show a drop in the concentration of the predator population at 8 hours (approximately 5 Log_10_ reduction) followed by recovery of *B. bacteriovorus* concentration and an associated delayed drop in the prey population at 120 hours. Due to the altered genetic background of HD100 mCherry (chromosomal integration of the mCherry gene) and associated biological strain expressing the mCherry protein, the dynamics of predation may be different to the wildtype, but the overall behaviour is the same (Fig. 8a). Therefore these HD100 mCherry results were not used for modelling purposes.

When predation events in serum were observed microscopically, we saw a rapid, early change in predator morphology. HD100 mCherry with typical vibroid morphology at 0 hours changed to a rounded morphology by two hours in serum with *K. pneumoniae* prey (Fig. 8b). Microscopic examples of fluorescent HD100 mCherry with altered morphologies are given in supplementary material Figure S3ab and the number of individual cells measured at each timepoint are provided in supplementary material Figure S4. The entire predator population began with a typical vibroid morphology (96.5% of all cells measured) but rapidly changed to a rounded morphology by two hours in serum (1.94% of all cells measured retained a vibroid morphology at 2 hours). The proportion of the predator population with a vibroid morphology remained low up to 8 hours (3.67%) before beginning to slowly recover from 24 hours onwards (Fig. 8b). This trend coincided with an increase in viable counts in the predator population (Fig. 8a), presumably due to successful predation. This change in predator morphology is likely to have a large impact on predation events, including attachment to prey.

The model fits the experimental data well using a reduced early attachment rate (*k*_1_^*early*^) and introducing a delay parameter, δ, to determine how long that reduced attachment continues. As can be seen from the experimental data (Fig. 5a,b), the length of the delay and the extent of the reduced attachment is variable between independent experiments in serum, and so studying the mean of all the experiments would be inappropriate as the smoothing effect would remove vital dynamics. For this reason, we began by fitting the parameters to the mean of experiments 1 and 2 only, which showed similar temporal dynamics in the experimental data, to obtain best-fit parameter values. We then set all the parameters to these best-fit values except *k*_1_^*early*^ and δ which we fitted individually to each independent experiment. Figure 6a shows model simulations using best fit parameters for the model plotted using the mean of experiments 1 and 2. This serum model simulation (Figure 6a,i) shows, in line with experimental results, the drop in prey concentration at approximately 48 hours followed by regrowth to a stable concentration of approximately 2 × 10^8^ cells/ml. *B. bacteriovorus* concentration in this simulation also compares well with the experimental data. Figure 6c shows the corresponding concentrations of predators attached to prey (PBD_O_), predators inside prey (PBD_I_) and inactivated predators (BD_I_) in the model simulation. These plots show that predation is halted until approximated 10 hours (as given by the delay parameter *δ*), then proceeds. Attachment rapidly declines after 56 hours as the prey become resistant to predation in the model (and apparently are predation resistant in our experimental data (Fig. 5a,b)). The model predicts a *B. bacteriovorus* replication time of 10 hours, which is longer than predation in buffer and is in line with estimates based on the rate of *B. bacteriovorus* increases in the experimental data from all biological repeats (Fig. 5).

The growth resources available to prey in the model have two sources, an initial serum component, which is rapidly depleted in the first hours of the simulation, and a predation-effected component which becomes significant in the system at approximately 50 hours as prey-lysis products start to accumulate, giving the high level of *K. pneumoniae* regrowth seen after 56 hours. Sensitivity analysis of the serum model (Supplementary material, Figure S5) at key points in the time course (early phase; lowest prey concentration stage and regrowth stage) highlights the multifactorial nature of the serum experimental system. Whilst the model is robust to changes in many parameters, it is most sensitive to changes in parameters relating to the death of *B. bacteriovorus* due to serum. In particular the prey concentration at 60 hours is by far the most sensitive to this, suggesting that changes in the ability of predator to resist the serum effects could have a large effect on the drop in prey numbers at this point.

**Figure 9:**
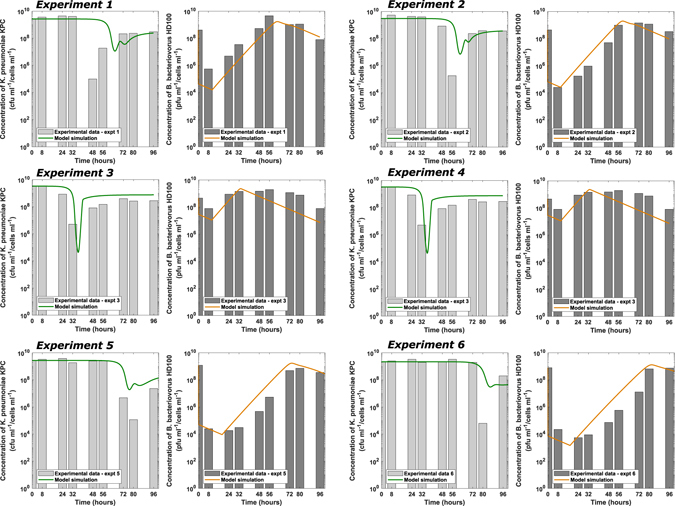
Model simulations of predation of *K. pneumoniae* by *B. bacteriovorus* in human serum, with varying levels delayed successful attachment, allows the model to fit each independent experiment (see Fig. 5 for experimental results).The delayed attachment parameter values for each experiment are k_1_^early^ = [1.433 × 10^−14^; 1.204 × 10^−14^; 3.999 × 10^−12^; 2.175 × 10^−11^_;_ 6.597 × 10^−16^; 2.304 × 10^−16^] and δ = [11.03; 10.29; 10.43; 10.55; 18.64; 17.49]. The remaining parameters which are fixed between experiments are given in Supplementary Table S2, in the supplementary material.

To validate the best fit parameters we use in the model we also repeated the parameter estimation using two other groups of experiments (experiments 3 and 4; experiments 5 and 6). In both cases the only significant difference between the parameters chosen was the early attachment rate *k*_1_^*early*^ (Supplementary material, Figure S6) as discussed above. Estimating the early attachment rate (*k*_1_^*early*^) and delay time (*δ*) individually showed us that the different behaviours observed experimentally can be substantially explained, and the model fitted to the all experimental data (Fig. 9) despite the complexity of the experimental data. The model suggests a small early attachment rate for all experiments with some variation in the extent of this and the time it takes to return to higher rates, hence free *B. bacteriovorus* get little protection from the serum at early timepoints and many are killed by serum antimicrobials and predator debris/predatory enzymes, with only a limited number of predators successfully entering prey at this stage.

Notably, a relatively small variation in the length of reduced attachment leads to much larger variation in the timing of the significant drop in prey numbers, this would support the view that the rounded predator cell majority are permanently prevented from entering prey, hence the long delay leads to reduced predator reproduction and it takes time to build up the progeny number to effectively then reduce *K. pneumoniae* numbers. A model with no early predator attachment to prey did not fit the data (not shown). The model does (Figure 6aii) predict an active but small predatory population at early timepoints (approximately 0.05% of the initial predator input at 2 hrs) and this correlated with a low percentage of vibroid *B. bacteriovorus* (1.94%) at the same timepoint in Fig. 8b).

**Figure 10:**
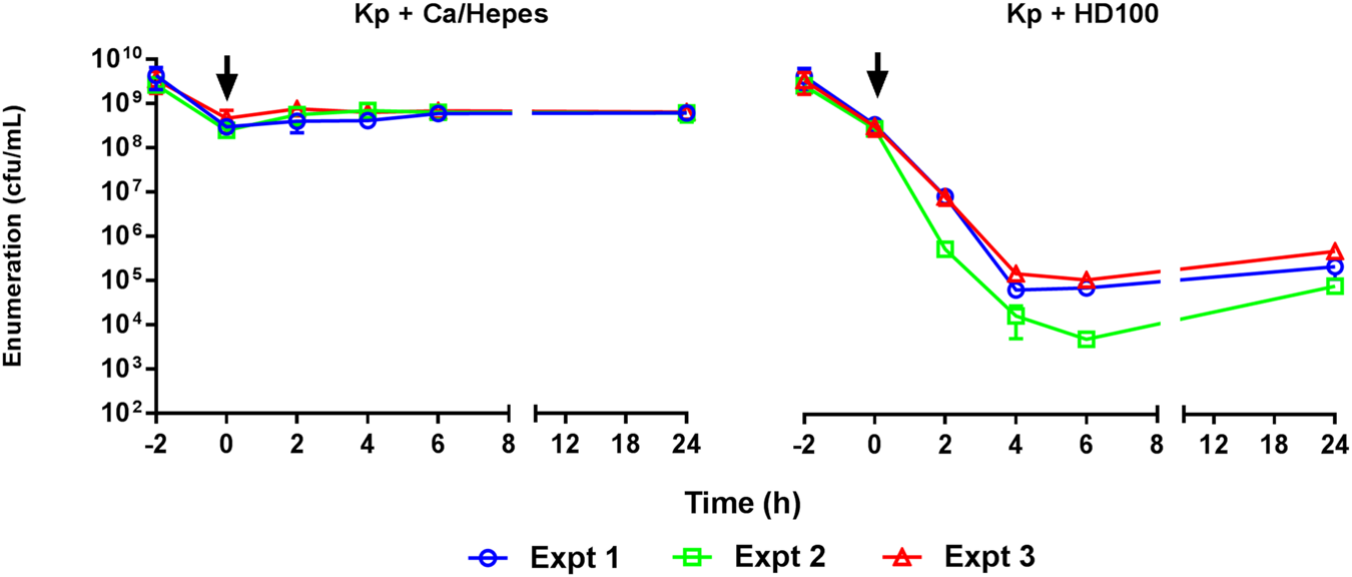
Predation in Ca/HEPES showing live bacterial numbers following pre-exposure of predator and prey to human serum. *B. bacteriovorus* HD100 and *K. pneumoniae* were exposed to human serum for two hours (t = −2 to 0 h). Predator and prey were then resuspended in Ca/HEPES and combined together in a predation assay and changes in the *K. pneumoniae* prey population enumerated periodically (Kp + HD100). As a control, *K. pneumoniae* prey previously exposed to human serum was resuspended in Ca/HEPES and enumerated periodically in the absence of *B. bacteriovorus* HD100 predator (Kp + Ca/HEPES). Arrows indicate the time at which bacteria were transferred from serum to buffer and subjected to respective treatment). *B. bacteriovorus* previously exposed to whole serum was added to *K. pneumoniae* to give a predator concentration at t = 0 h of approximately 5 × 10^8^ pfu/ml.

In order to investigate the potential for serum derived components to bind to the surface of predator or prey and possibly halt predation, we separately exposed *B. bacteriovorus* and *K. pneumoniae* to whole human serum, prior to subsequent predation in Ca/HEPES buffer (Fig. 10). Pre-exposure of both predator and prey separately to whole human serum for two hours was found to have no subsequent delaying effect on predation once *B. bacteriovorus* and *K. pneumoniae* were removed from serum and combined in Ca/HEPES buffer. Indeed, predation of *K. pneumoniae* (mean concentration at 0 hours 3.17 × 10^8^ cfu/ml) by *B. bacteriovorus* (mean concentration at 0 hours 3.65 × 10^8^ cfu/ml) following exposure to human serum proceeded in an almost identical fashion to that reported earlier in Ca/HEPES buffer (Fig. 10 vs Fig. 2a) with an approximate five Log_10_ drop in prey concentration by four hours incubation time. These results lead us to conclude that a binding of serum components, or early predator debris, to the cell surface of either *K. pneumoniae* or *B. bacteriovorus, (*that could inhibit attachment of active predators*)*, is either highly reversible on washing in buffer, or unlikely to be the cause of the delay in predation we observe in our serum predation assays.

### Changes in serum complement activity

It is not possible to measure experimentally (from a predator-prey mixture) all the antimicrobial components of serum, but one of the major antibacterial factors in serum that we could readily measure was complement through the classical pathway, using a haemolysis assay with predators and prey alone or in combination (Fig. 7a)^30, 31^. Additionally, changes in viability of the bacterial populations incubated in serum were enumerated over the 96 hour time period (Fig. 7b). Serum incubated in the presence of *B. bacteriovorus* alone, *K. pneumoniae* alone or in the presence of both predator and prey was depleted of classical complement pathway activity by 4 hours, the earliest time point measured. This experimental result fits with our mathematical model, which predicts the depletion of serum antimicrobial activity in less than 1 hour. This rapid loss of complement activity highlights one of the limitations of using a closed experimental system and should be considered when extrapolating results to some *in vivo* scenarios such as the circulatory system^38^, but is of relevance to isolated wound sites. It is possible that in a more refreshed system, where serum is replenished by bodily circulation that once prey numbers are reduced below a threshold level by *B. bacteriovorus* predation the antimicrobial activity of the serum, in combination with leukocytes, may clear the pathogen and the pathogen regrowth we observe in our closed model systems would not occur. We have established that we have a model that describes the additional complexity of predation in serum and accurately fits the extensive experimental results. Moreover, the modelling has guided experimentation to clarify the mechanisms involved in predator-prey-serum interactions.

## Discussion

Antimicrobial resistance has emerged as a global health issue and has stimulated us to test and model how predatory *B. bacteriovorus* prey upon *K. pneumoniae* in this study. Multidrug resistant Gram-negative bacterial infections, especially those as a result of so-called ESKAPE pathogens^39^, including *K. pneumoniae*, are a real problem in developing and developed countries and in the healthcare setting^13^. Many of these infections include multidrug-resistant strains, at different bodily sites, designated currently ‘untreatable’ using conventional antibiotics^40, 41^. As a response, alternatives to antibiotics are being sought^42, 43^. The use of predatory bacteria, such as *B. bacteriovorus*, as “living antibiotics” is an attractive prospect but it is crucial to investigate *Bdellovibrio*-pathogen interactions and kinetics of predation in host-relevant environments, and to be able to predict the effects of predator doses, if they are to be used as a therapeutic agents. In light of this, we chose to model pathogen-predation in buffer compared to that in human serum.

Research to date modelling and quantifying the activity of predatory bacteria has focused mostly on laboratory strains of prey bacteria in ideal (buffer or growth media) conditions. Separate research has tested the effects of pathogen treatments with predators *in vivo* without modelling. We need to improve our understanding of host relevant conditions under which *B. bacteriovorus* can prey on clinical, antibiotic-resistant strains of bacteria that cause untreatable infections. Although promising steps have been taken towards this with *in vivo* experimental studies of *B. bacteriovorus* as a therapeutic^28, 44^, including leukocyte interactions in Willis and co-workers, these experiments did not investigate underlying serum antibacterial mechanisms that may affect the dynamics of predation in a therapeutic setting. To address this, we set out to develop a model that captures the complexities of human serum and could therefore inform on predation dynamics in this setting and guide experimentation.

We have shown that *B. bacteriovorus* HD100 can effectively prey in both buffer and human serum on a clinical isolate of *K. pneumoniae* KPC, a major cause of carbapenem-resistant infections and problematic as a carrier status in patients^45^. We developed a mathematical model which fits the experimental data closely and we used this model to explore the underlying dynamics of predation. Through this interdisciplinary approach, we have shown that the interactions of this predator and pathogen in the human serum environment, and their responses to it are markedly different to that seen in buffer. These differences are indicative of the inherent problems of simplifying clinical processes to laboratory standards.

The mathematical model of the buffer-based predation experiments included terms relating to predation, prey growth and previously proposed “plastic resistance” phenotypes^17^, gave a good fit to the experimental data for both predator and prey concentrations over a 96 hour period and was validated with an independent data set. The parameter estimates for predator internalisation and time to predator-progeny release in buffer were within the range of observed times in *E. coil* ^35^, and the estimated natural predator death rate was also in line with previous studies in buffer^14^. These factors lead us to believe we have a functional mechanistic model of *B. bacteriovorus* predation that includes the dominant mechanisms.

This paper shows for the first time that predation of a specific clinical pathogen by a predator in serum is markedly different to predation in buffer, and through the development of mathematical models directing further experimental validation, where experimentally tractable, we have addressed why this is the case. We show that, in the case of the predation in serum model, reduced initial predator attachment for a period up to 19 hours, compared to the buffer model, makes it possible for the model to fit the serum-predation experimental results. Our model assumes that every attachment event results in successful predation, therefore the term “reduced attachment” as used here, represents an increase in unsuccessful predator-prey attachment events that do not result in predation or simply less frequent attachment events occurring. To determine the cause of reduced early predator attachment, as suggested by the model, serum pre-incubation of predators or prey separately, prior to predation tests in buffer, were performed. These experiments suggested that the delay in predation observed was not as a result of direct binding of serum components to either the predator or prey cell surface, blocking attachment, as predation following exposure to human serum proceeded in an almost identical fashion to that reported earlier in Ca/HEPES buffer. Nor were the predators or prey irreversibly changed by the serum pre-treatment.

Serum is a complex mixture which is not amenable to microscopic work with small, unlabelled, predatory bacteria. However, the need to visualise predators during this delayed predation period led us to make additional microscopic observations of predation in serum using fluorescent HD100 mCherry predators. This showed that *B. bacteriovorus* rapidly changed from a typical vibroid morphology to a rounded morphology on initial contact with serum. At population level this altered morphology was temporary and predators were seen to have returned back to vibroid over the time course of the study. Vibroid morphology was associated with an increase in concentration of the predator population, presumably due to successful predation. Predator rounding may be more likely in a predator that has bound to and sensed prey but proving this experimentally is beyond the scope of this study. These results do suggest that preconditioning *B. bacteriovorus* to human serum is relevant for any future therapeutic use.

The detection of the predation delay in serum and directed experimentation, described above, emphasises the utility of our mathematical model, it provides insights into the population dynamics of predation that are difficult or impossible to determine experimentally. Predation events, such as attachment, are short lived and difficult to measure accurately, en masse, by direct microscopic observation, a task which becomes even more arduous in a complex granular medium such as serum. We therefore hypothesize that the delay in predation we observe in serum is a result of reduced successful attachment events arising from a change in predator morphology, which we observe experimentally. This morphology returns to vibroid over time, correlating with increasing *B. bacteriovorus* cell numbers after rare predation events have allowed predator replication. The observed delay in predation should be further explored experimentally, and studies investigating the transcriptional response of both predator and prey adapting to a serum environment are underway and may provide answers to this complex issue, but are beyond the scope of this work.

After accumulation of predators via rounds of replication there is a significant drop in the *K. pneumoniae* prey numbers but later there is a period of regrowth (Fig. 5). We make the assumption that prey cells accumulating in a serum-based population are becoming resistant to predation following initial prey bursting and exit in their neighbourhood. We postulate that this resistance is a plastic phenotypic resistance, as this has been reported previously for the predation of another gammaproteobacterium, *Erwinia carotovora* in buffer^17^. It should be stressed that this plastic resistance is believed to transient in nature rather than due to genetic mutation of the prey. Shemesh and Jurkevitch hypothesised that this phenotypic resistance may be due to modification they describe as “hardening of the prey cell wall” resulting from the release of low levels of lytic enzymes by predators during ineffective prey cell encounters. In our model, the development of prey phenotypic resistance in serum is dependent upon the amount of prey cell lysis resulting from predation. Lysed prey will release “waste”:- including a mixture of both intracellular prey enzymes and predator lytic enzymes, produced to effect bdelloplast exit. These free lytic enzymes may act on neighbouring cells, possibly resulting in the “hardening effect” described, or there may be other “anti-predation” modifications which await characterisation. This explanation would support the work of Shemesh and Jurkevitch, that predatory enzymes may play an indirect role in producing a transiently resistant prey population^17^. Alternatively, this “predation-waste”, containing the remnants of prey cell membranes and walls left over from lysis^46^, may act as a physical barrier to attachment. We note, however, that the formulation of the model allows flexibility such that the model is not restricted to a particular underlying biological mechanism(s) for this prey regrowth after predation. In a replenished circulatory system, *in vivo*, rather than the batch system we tested, we would not expect such waste accumulation and so such a “resistant” prey response may not be seen in privileged or minimally perfused body sites. The zebrafish infection study of Willis *et al*.^28^ may be a relevant example.

Predator-prey-serum interaction kinetics are important if *B. bacteriovorus* are ever to be used as topical (e.g. on tracheotomies), ingested (to attempt gut clearance for patients who are carriers), applied to wounds (surgically or environmentally caused) or inhaled (for lung infection) therapies for human *Klebsiella* infections. Recently Shatzkes *et al*.^44^ have highlighted the potential of one of these approaches, showing that repeated intranasal administration of. *B. bacteriovorus* 109 J can reduce the bacterial burden of *K. pneumoniae* in rat lungs. Their study was able to measure prey, but not predator, viability over a 24 hour period reporting a reduction of *K. pneumoniae* prey in the majority of animals.

The extended live predator and prey count data in our study emphasise the importance of building upon such studies^44^ to understand the dynamics of predation beyond any initial reduction in the prey population. The prey regrowth we observe in buffer and serum, presumably due to plastic resistance, could impede convalescence after *B. bacteriovorus* treatment and is important to address, hence our inclusion of a resistance mechanism in the model. Whilst the potential to treat an initial *K. pneumoniae* infection with *B. bacteriovorus* has been established^44^, preventing pathogen regrowth is just as crucial and needs further research. Recently, Willis *et al*.^28^ demonstrated for the first time that injected *B. bacteriovorus* can be used to cure an otherwise lethal bacterial infection in living animals. They found that the synergistic action of the innate immune system with the predatory bacteria were responsible for reduced pathogen numbers and increased survival in zebrafish larvae. Our serum predation modelling is an important precursor to a further study including leukocytes and as a “3Rs” strategy to reduce the use of unnecessary animals in medical research.

The approach we take here, combining modelling with experimental verification of growth response by a clinical pathogen in human serum, shows that pathogen regrowth after predation can be modelled. This encourages future quantitative research in more complex bodily settings, such as wounds, with innate immune cells acting also. This approach may aid dosing and viable predator-persistence considerations for more systemic studies such as the bloodstream injections of predators recently published by Shatzkes *et al*.^38^.

Our work here and future models will assist the design of a treatment regime aimed at preventing such regrowth, such as exploring whether multiple doses of *B. bacteriovorus* could synergise with the immune system to clear an infection.

In summary, in this work we have shown for the first time that *B. bacteriovorus* can effectively prey on the antibiotic-resistant, human-pathogen *K. pneumoniae* KPC in human serum, as well as buffer. There can be later regrowth of the pathogen, post-predation, which needs to be researched further. We have shown that the dynamics of predation in human serum are complex and differ significantly from the comparatively simple dynamics of predation in buffer. The synergy of mathematical modelling and experimental science has provided new theories for the mechanisms behind these differences. These insights guide future experimentation to mitigate delayed predation in serum and prey regrowth, for efficient therapeutic administration of *B. bacteriovorus* in serum-rich settings, such as infected wounds. Also a study of how, or if, predators and prey secrete compounds that change serum composition will be valuable. Future strategies to produce the most active predator doses may come from pre-adaptation of predators in serum prior to administration, or the development of serum resistant *B. bacteriovorus* strains through forced evolution. This study has highlighted the importance of using realistic, bodily-derived media, extended time-course studies and mathematical modelling as we take steps towards validating predatory bacteria as living antibiotics.

## Electronic supplementary material

Supplmentary Information
