# Supplementary material for: Measuring and modelling the response of Klebsiella pneumoniae KPC prey to Bdellovibrio bacteriovorus predation, in human serum and defined buffer
Source: Sci Rep. 2017 Aug 21;7:8329. doi: 10.1038/s41598-017-08060-4 (PMC5567095; doi:10.1038/s41598-017-08060-4)
Supplement: Supplementary file 1 — Supplmentary Information [file 41598_2017_8060_MOESM1_ESM.pdf]

Supplementary Material - Measuring and modelling the response of *Klebsiella pneumoniae* KPC prey to *Bdellovibrio bacteriovorus* predation, in human serum and defined buffer

Authors: Michelle Baker[1ab], David Negus[1a], Dhaarini Raghunathan[a], Paul Radford[a], Chris Moore[a], Gemma Clark[c], Mathew Diggle[c], Jess Tyson[a], Jamie Twycross[b], R. Elizabeth Sockett[a]*

[1] ***Joint first authors*** *****Corresponding author***

Mathematical modelling

The predation of *K. pnuemoniae KPC* by *B. bacteriovorus* HD100 was modelled in both buffer and human serum. Since laboratory culture involves continuous shaking a relatively homogeneous environment is established hence, ordinary differential equations (ODEs) were used. The models have been developed based on the biological mechanisms involved in the predatory cycle and have been influenced by the work of several laboratories[1-5].

Buffer-based model

The Ca/HEPES buffer model has eight variables: free *B. bacteriovorus* predators (BD_F), uninfected predation-susceptible *K. pneumoniae* prey (P_S), uninfected predation-resistant *K. pneumoniae* prey (P_R), invaded prey with predator attached outside (PBD_O), invaded prey with predator on the inside (PBD_I), inactivated predator (BD_I), nutritious predation-derived waste (W_N) and non-degradable predation-derived waste (W_M). The interactions between these variables is shown diagrammatically in Figure 1 and described by the system of equations,

$$\frac{d[BD_F]}{dt} = k_3\rho[PBD_I] + k_4[BD_I] - k_1[BD_F][P_S] - k_1[BD_F][PBD_O] - \lambda[BD_F] \qquad (1)$$

$$\frac{d[P_S]}{dt} = \frac{V_{max}^{w}[W_N]}{s_w+[W_N]}[P_S] - k_1[BD_F][P_S] - f[W_M][P_S] \quad (2)$$

$$\frac{d[P_R]}{dt} = \frac{V_{max}^{w}[W_N]}{s_w+[W_N]}[P_R] + f[W_M][P_S] \quad (3)$$

$$\frac{d[PBD_O]}{dt} = k_1[BD_F][P_S] - k_2[PBD_O] \quad (4)$$

$$\frac{d[PBD_I]}{dt} = k_2[PBD_O] - k_3[PBD_I] \quad (5)$$

$$\frac{d[BD_I]}{dt} = k_1[BD_F][PBD_O] - k_4[BD_I] \quad (6)$$

$$\frac{d[W_N]}{dt} = d_P k_3[PBD_I] - E_w \frac{V_{max}^{w}[W_N]}{s_w+[W_N]}([P_S] + [P_R]) \quad (7)$$

$$\frac{d[W_M]}{dt} = d_P k_3[PBD_I] \quad (8)$$

The free *B. bacteriovorus* (BD_F) population is the free-swimming *B. bacteriovorus* population. Predators leave this population by attaching to prey, either irreversibly or temporarily, or by death at the natural death rate, λ. Predators join the population by replication within prey and by released attachments with invaded prey. Prey independent growth of the predatory bacteria has been neglected from the model as these have been shown experimentally (unpublished results) to be at negligible levels within the media and the timescale we are concerned with (up to 120 hours). Equation (1) governs the change in concentration of BD_F where k_1 is the predator-pathogen binding rate, k_3 is the predator reproduction rate, k_4 is the predator prey detachment rate and ρ is the predator progeny number.

Resistance to *B. bacteriovorus* predation is not fully understood at present, but a 'plastic phenotypic', non-inherited resistance effect has been observed experimentally[6]. To account for this in the model there are two populations of uninfected prey, susceptible and resistant (equations (2) and (3)). In all simulations the model is initialised with only susceptible populations. Susceptible uninfected prey can then become resistant at a rate that is a function of the amount of non-degradable waste in the system, which itself is dependent

upon successful predation. This leads to an increasing fraction of the prey population becoming resistant over time. This resistance could be a result of increasing concentrations of non-degradable waste products (including cell wall/membrane fragments) diffusing through the system and impeding predator-prey interactions. However, since the resistance term used is relatively general it also allows for other mechanisms of plastic resistance.

Uninfected predation-susceptible *K. pneumoniae* prey (P_S) dynamics (equation 2) include bacterial growth in the presence of a growth nutrient, W_N released from dead prey cells. The per capita growth rate ψ_w is a Hill function where V_{max}^{w} is the maximum growth rate in the presence of unlimited resources and s_w is the Michaelis constant (i.e. the concentration of W_N resulting in ψ_w = 50% of V_{max}^{w}). The Hill function is dependent on W_N, hence as the growth resources are depleted the growth rate decreases. The dynamics of uninfected predation-resistant *K. pneumoniae* prey (P_R) are similar but do not include any predation terms (equation 3).

Invasion of prey is not instantaneous: *B. bacteriovorus* first attach to the outside of the prey and spend some time recognising and then modifying the prey cell wall to create a hole to move though, we include this time implicitly within the attachment rate parameter. Time is also taken for the predator to move through the cell wall of the bacteria during which time other predators may attempt to invade the same prey cell[7], for this reason, there are two populations of invaded prey: prey with predators attached to the outside, which are in the process of moving through the wall (PBD_O) and prey with predator completely on the inside (PBD_I). The source of the PBD_O population depends on the interaction of BD_F and P. Predators are then internalised into the prey at the prey internalisation rate, k_2, leading to movement from the PBD_O population to the PBD_I population. The PBD_I population decreases only because of predator replication. The final bacterial variable in the model is BD_I, which represents predators that are trying to invade already invaded prey, but will ultimately be unsuccessful and return to the population of free predators[7].

Ca/HEPES buffer is free of any growth nutrients and is an environment in which prey alone would persist at stable levels over the experimental time duration. As predation occurs and prey cells are lysed it is assumed that this leads to “waste products” diffusing into the buffer and we put this waste into two categories. Nutritious waste is waste which can be consumed by the prey bacteria and used as a growth resource, resulting in an increased prey population. Non-degradable waste is any other waste products of predation which cannot then subsequently be consumed by prey and instead obstruct predator-prey interactions. This waste will monotonically increase over the course of a simulation. These two types of waste are described by equations 7 and 8 where d_P is the dimensionless waste fraction generated from lysis and E_w is the efficiency of waste conversion.

Serum Model Extension

To model the predation system in human serum the model was extended to include two serum specific variables, serum growth nutrient (*R*) and serum antimicrobials (*I*). Serum growth nutrient is considered to decrease as it is used by prey cells, and is not replenished. The per capita growth function is a Hill functions, as in the case of W_N. Serum antimicrobials in the model cause the death of both predators (BD_F) and prey (P_S and P_R) in the model, using up *I* in the process. The serum antimicrobial activity is considered to be exponentially decaying over time even in the absence of bacteria. Interactions between prey and serum antimicrobials depend on a fractional index, *n*, to account for the non-linear interactions as a result of multiple pathways for serum antimicrobial activity. A fractional index for predator-serum interactions was not required as results were only negligibly improved.

Additional terms were also added to the *B. bacteriovorus* and prey dynamics (equations 1-3). This is to account for serum-mediated cell death and serum-mediated prey growth (as there is added potential nutritional content in serum compared to buffer). A time-dependent predator removal function was added to equation 1 to account for the death of *B. bacteriovorus* as a result of increasing debris levels from predator death (discussed in

Results). Additional terms were added to equations 2 and 3 to account for prey interactions with the serum, as discussed. For the predation attachment (k_1) rate, two different rates are modelled (equations 19) with a switch at time δ. This allows for an initial delay in successful (prey invasive) predator attachment, which is seen in microscopy images and reflected in the experimental results. This reduced attachment is discussed extensively in the Results section of the main text. Hence, the full serum model is,

$$\frac{d[BD_F]}{dt} = k_3\rho[PBD_I] + k_4[BD_I] - k_1[BD_F][P_S] - k_1[BD_F][PBD_O] - a_{BD}[I][BD_F] - \left(\frac{d_{max}^{BD}}{1+he^{-BD_D}} + d_{min}^{BD}\right)[BD_F]t - \lambda[BD_F] \quad (9)$$

$$\frac{d[P_S]}{dt} = \frac{V_{max}[R]}{s+[R]}[P_S] + \frac{V_{max}^{w}[W_N]}{s_w+[W_N]}[P_S] - k_1[BD_F][P_S] - a_P[I][P_S] - f[W_M][P_S] \quad (10)$$

$$\frac{d[P_R]}{dt} = \frac{V_{max}[R]}{s+[R]}[P_R] + \frac{V_{max}^{w}[W_N]}{s_w+[W_N]}[P_R] - a_P[I][P_R] + f[W_M][P_S] \quad (11)$$

$$\frac{d[PBD_O]}{dt} = k_1[BD_F][P_S] - k_2[PBD_O] \quad (12)$$

$$\frac{d[PBD_I]}{dt} = k_2[PBD_O] - k_3[PBD_I] \quad (13)$$

$$\frac{d[BD_I]}{dt} = k_1[BD_F][PBD_O] - k_4[BD_I] \quad (14)$$

$$\frac{d[W_N]}{dt} = d_P k_3[PBD_I] - E_w\frac{V_{max}^{w}[W_N]}{s_w+[W_N]}([P_S] + [P_R]) \quad (15)$$

$$\frac{d[W_M]}{dt} = d_P k_3[PBD_I] \quad (16)$$

$$\frac{d[R]}{dt} = -E\frac{V_{max}[R]}{s+[R]}([P_S] + [P_R]) \quad (17)$$

$$\frac{d[I]}{dt} = -d_I I - c_P[I]([P_S] + [P_R])^n - c_{BD}[I][BD_F] \quad (18)$$

where,

$$k_1 = \begin{cases} k_1^{early} & t \leq \delta \\ k_1^{late} & t > \delta \end{cases} \quad (19)$$

$$BD_D = a_{BD}[I][BD_F] + \left(\frac{d_{max}^{BD}}{1+he^{-BD_D}} + d_{min}^{BD}\right)[BD_F]t + \lambda[BD_F] \quad (20)$$

Reporting variables

To compare the model to the experimental data we need to report variables that match the cell types that would form colonies / plaques during enumeration. For *K. pneumoniae* we know that cell death occurs during the rounding stage of Bdelloplast formation [8], which in terms of the model will be at some point whilst the prey are in the PDB_I stage. Hence we calculate the concentration of prey colonies as,

$$\text{PreyCFU} = P_S + P_R + PBD_O + \nu PBD_I,$$

where *v* is a dimensionless term representing the proportion of invaded prey cells that are still reportable. We estimate this parameter since it has not been determined experimentally.

For the *B. bacteriovorus* concentration we expect that once a predator is attached to its prey the predation process will continue on the plaque plate. Hence we calculate the concentration of predator plaques as,

$$\text{PredatorPFU} = BD_F + PBD_O + PBD_I + BD_I.$$

Parameter Estimation

The model was simulated and parameters were fitted using MATLAB [9]. Since the system is analytically intractable and stiff the model was solved numerically using the *ode15s* routine. The parameters were estimated using the non-linear least squares routine *lsqnonlin*, part of the optimization toolbox in MATLAB. This routine uses a trust reflective algorithm for optimization. To fit parameters, 10000 random parameter sets were generated and each set was used as an initial guess to estimate the optimal local parameters using the least squares routine. The residual at each point was calculated as the sum of the logged differences

between the observed and simulated data. The best set of optimal parameters were then selected from the results using the minimum 2-norm of the residual.

Parameter Validation

To confirm the validity of the parameters estimated from the model we searched the literature for relevant parameter estimates. Our estimated value for λ, the natural predator death rate, is in line with predator death rates previously reported from both experimental and computational work [3,10]. We estimate the V_{max} generation time for *K. pneumoniae* in serum to be approximately 1 hour and 40 minutes. Whilst no studies of other *Klebsiella* growth rates in serum have been reported, to our knowledge, this estimate fits our experimental controls well and fits with reported *K. pneumoniae* growth in batch on a synthetic medium[11] and in glucose limited chemostats[12], and is as expected slower than *Klebsiella* growth in nutrient broth[13]. Our estimate for the predator internalisation rate, k_2, which represents the time taken for the predator to move through the prey cell wall after attachment is in line with previous published experimental measurements of internalisation through *Escherichia coli*[7]. We measured the predator progeny number, ρ, experimentally in buffer (procedure and results detailed below). Comparison of size differences between prey in buffer and serum would suggest that the number of progeny might be lower in serum experiments, however parameter estimation of this parameter gave a value of 3.52, not significantly different from that measured in buffer (3.85 $\pm$ 0.89, see next section), hence we chose to use the measured value and reduce the number of parameters estimated in the serum model. All estimated parameters have been validated against the experimental data sets used for estimation, and fit the data to within experimental error bars. Many parameters have also been independently validated with unseen experimental data (i.e. data that has not been used to estimate the parameters). The data used for estimation and validation procedure is described in Supplementary Table 3. For predation in buffer we simulate an independent set of experimental data using the parameters we estimated. Figure S7 shows that the model gives a good qualitative fit to this data, although there may be a shift time shift

in the onset of predation compared to the experimental data. For predation in human serum as a validation step, we ran two further independent parameter estimation procedures based on experiment 3 and 4, then 5 and 6. We grouped the data in this way so that the temporal differences in the results did not remove the overall dynamics when taking an average. Supplementary Figure S6 shows a comparison of the best fit parameters from each of the parameter estimation procedures. The only major differences in the values are in the parameters k_1^{early} and δ, the parameters that we believe to be key to the variability in the experimental results. All other parameter values are consistent with all three sets of experimental data, confirming the robustness of the model and parameter fitting technique. The individual fitting of the all six independent biological repeats to the best fit parameters generated from only experiments 1 and 2, and only changes to two of the 27 parameters, gives further validation of the model robustness to variation. As an additional test, we fitted the mean of a further three independent experiments where the early viable counts of *B. bacteriovorus* dropped below the limits of detection and a significant drop in prey numbers is not seen within the experimental time course (Supplementary Figure S8). The model was able to fit this data well, and predicts late predation events giving a significant drop in prey numbers followed by regrowth would have been seen at approximately 110 hours had the experimental time course been longer.

Predation burst size of *K. pneumoniae*: The number of *B. bacteriovorus* HD100 progeny released from each *K. pneumoniae* prey was measured by examination of light microscopy videos for a sample of 143 successful predations, complied from two biological repeats. Supplementary Figure S1 shows the distribution of burst sizes, with a mean of 3.85 (±0.89) and a median burst size of 4. On visual examination the number of *B. bacteriovorus* progeny appears to correlate to the size of the *K. pneumoniae* prey. This is in line with previous observations of Kessel and Shilo [14] of predation on *Escherichia coli*. We use the mean progeny number of 3.85 for both the buffer and serum modelling presented in this paper, as

estimation of *B. bacteriovorus* progeny for predation in serum gave a result within the error of this experimentally measured value.

Supplementary Figure S1: Histogram showing the number of predatory progeny released from a sample (n = 143) of *K. pneumoniae* KPC predation by *B. bacteriovorus* HD100 in Ca/HEPES buffer. The mean burst size is 3.85±0.89 and this value is used in all model simulations. The standard deviation was 0.89. Data was collated from 2 biological repeats (n = 119 and 24).

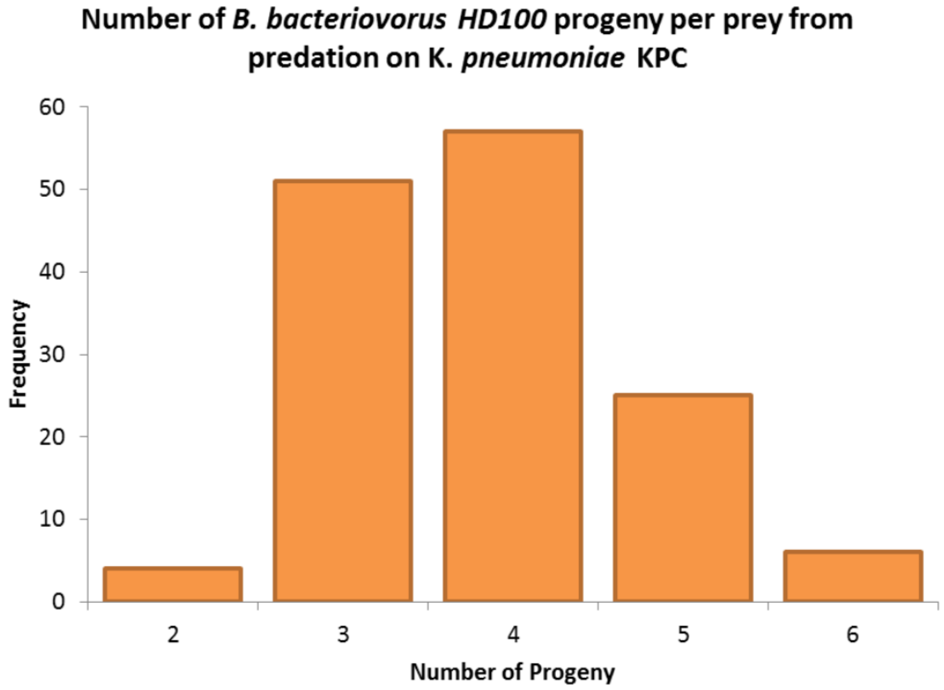

Supplementary Figure S2: Microscopic analysis of early *B. bacteriovorus* predation of *K. pneumoniae* in Ca/Hepes buffer. Predation assay was performed as described in materials and methods and aliquots were periodically withdrawn and placed on an Ca/HEPES agarose pad for microscopy. *K. pneumoniae* prey were imaged by phase contrast and mCherry expressing *B. bacteriovorus* predators were imaged in the fluorescent channel. Predator, prey and their interactions were counted manually in ImageJ software (Bd = *Bdellovibrio bacteriovorus*).

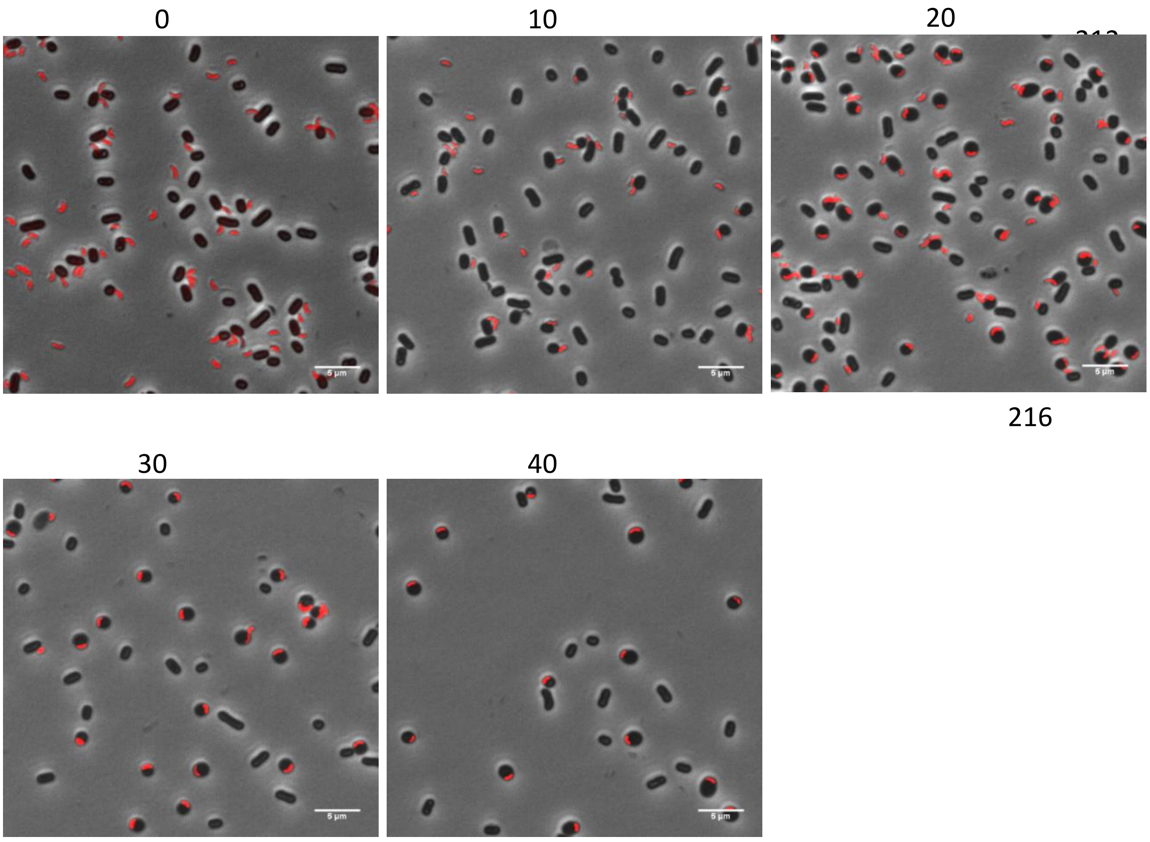

	Total Prey (n)	Total Bd (n)	Free Bd (n)	Attached Bd (n)	Internal Bd (n)	Free Bd (%)	Attached Bd (%)	Internal Bd (%)
0m	57	45	27	18	0	60	40	0
10m	65	39	11	28	0	28	72	0
20m	101	84	10	33	41	12	39	49
30m	43	25	0	4	21	0	16	84
40m	29	14	0	0	14	0	0	100

Supplementary Figure S3: Fluorescence microscopy of predation in serum at 0 hours (a) or 2 hours (b). *K. pneumoniae* prey were imaged by phase contrast and mCherry expressing *B. bacteriovorus* predators were imaged in the fluorescent channel. Fluorescent predators can be seen to change from a vibroid morphology to round. Expanded images are at 3X magnification of selected area.

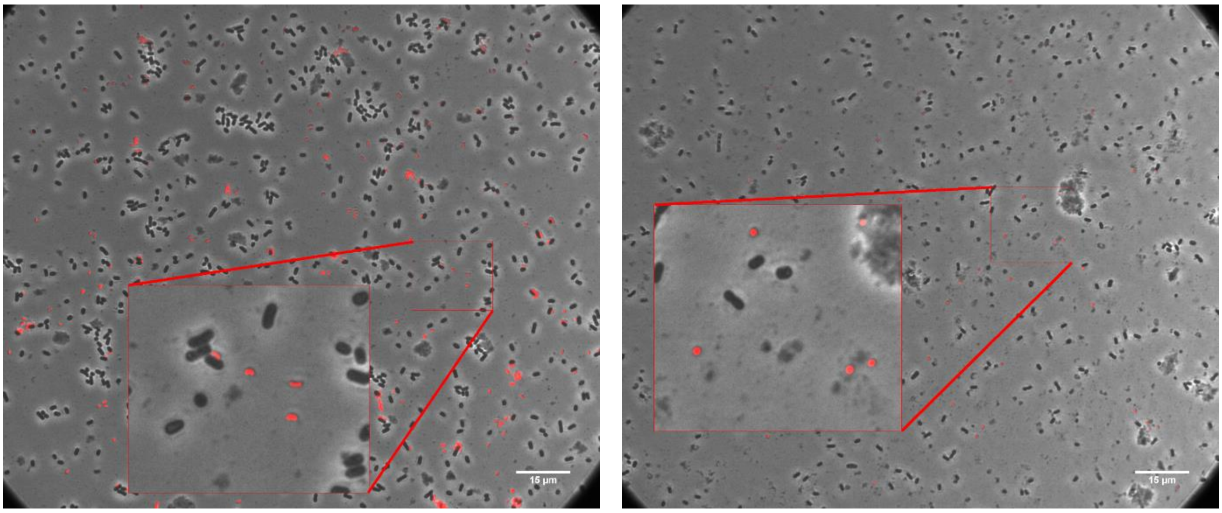

Supplementary Figure S4: MicrobeJ software was used to automatically detect mCherry tagged fluorescent *B. bacteriovorus* cells and measure the roundness (4 × area / (π × major axis2) of each individual cell. A roundness cut off of 0.65 was selected to distinguish between cells with typical vibroid morphology (roundness score <0.65) and those with an abnormal round morphology (roundness score ≥0.65). Example images of typical *B. bacteriovorus* cells imaged in the fluorescent channel and their respective roundness score are given below the table. Bd = *Bdellovibrio bacteriovorus*.

Time (h)	Total Bd Counted	Number of Bd with roundness equal or >0.65 (round)	Number of Bd with roundness <0.65 (vibroid)	% Round	% Vibroid
0	1026	36	990	3.5	96.5
2	310	304	6	98.06	1.94
4	264	243	21	92.05	7.95
6	357	345	12	96.64	3.36
8	109	105	4	96.33	3.67
24	223	159	64	71.3	28.7
32	146	113	33	77.4	22.6
48	259	204	55	78.76	21.24
56	340	261	79	76.76	23.24
72	441	209	232	47.4	52.6
80	328	157	171	47.87	52.13
96	415	204	211	49.16	50.84
104	990	417	573	42.12	57.88
120	677	180	497	26.59	73.41

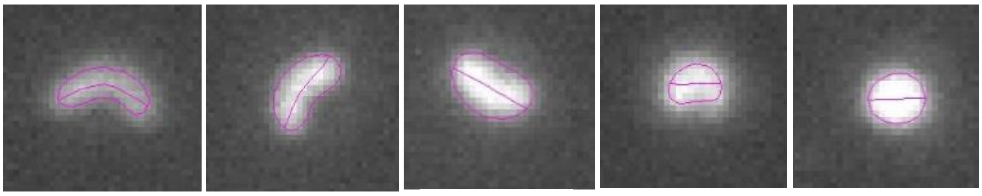

0.4 roundness 0.5 roundness 0.6 roundness 0.65 roundness 0.75 roundness

Supplementary Figure S5: One-at-a-time sensitivity analysis of a ±10% change in each parameter at three different time points in the serum model. The time points have been selected at key stages in the experimental time course (i.e. early reduced attachment, significant prey depletion and prey regrowth). The variation in parameter sensitivity at different times reflects the different mechanisms dominating predator and prey interactions at those times. The sensitivity coefficient is scaled such that a value of ±1 represents a ±10% change in concentration as a result of a ±10% change in parameter value. Parameter values detailed in Supplementary Table S2.

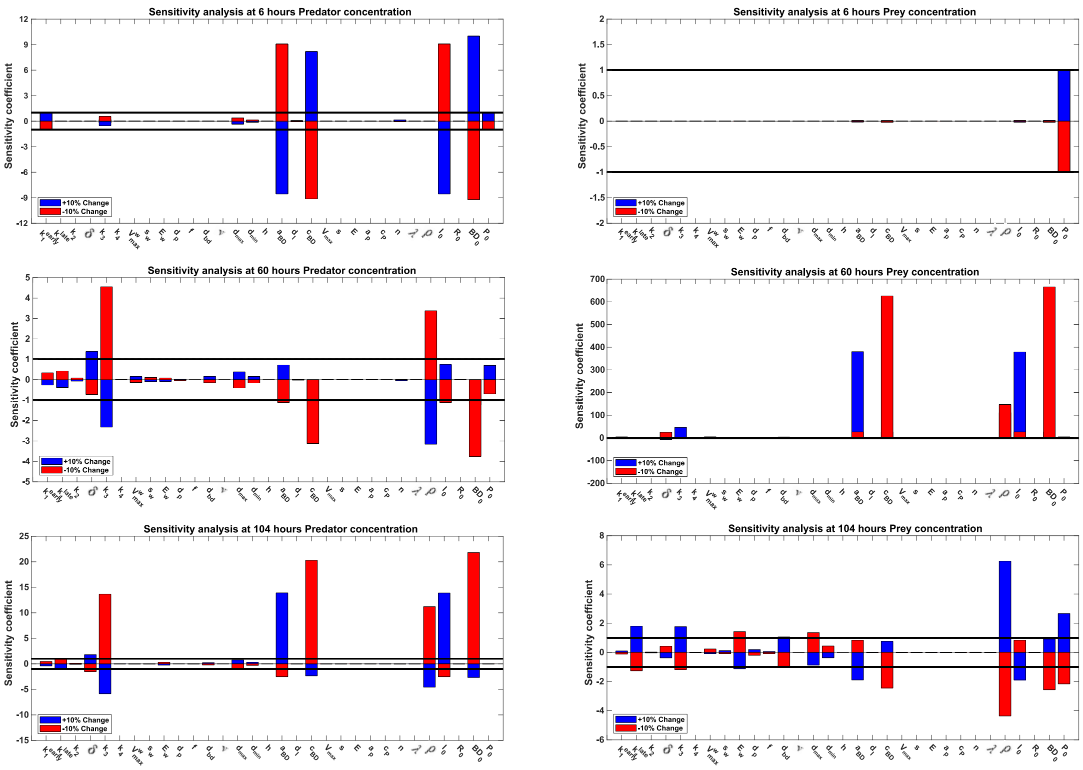

Supplementary Figure S6: Bar charts show parameter values estimated for the serum model using 3 independent data sets. The chart shows the consistency of the parameter estimation with only one parameter showing a large difference, which we propose has a biological mechanism, as discussed in the manuscript.

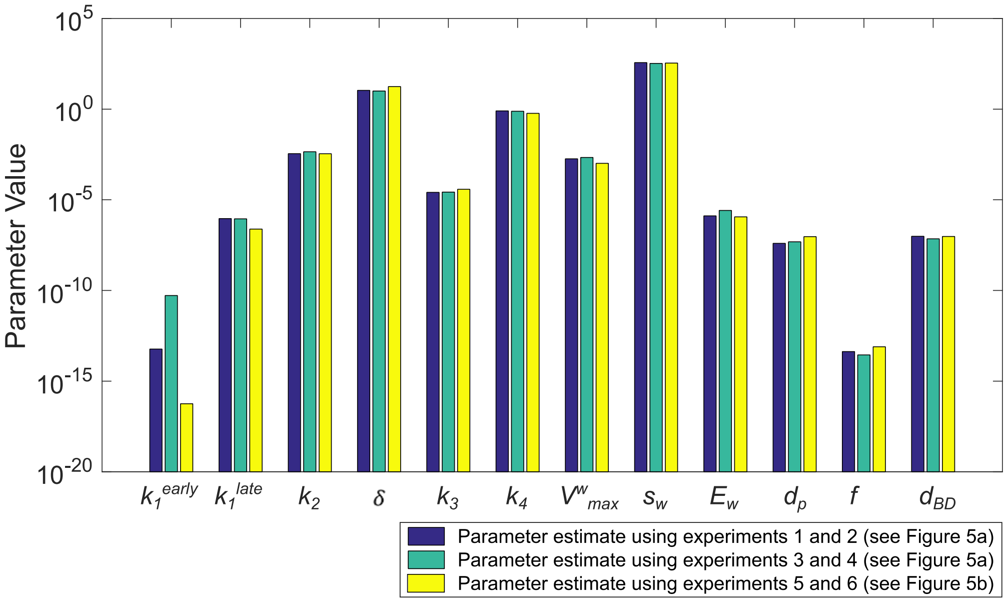

Supplementary Figure S7: Testing of the buffer model with additional experimental data validates the estimated parameters. The model simulation using initial *K. pneumoniae* and *B. bacteriovorus* concentrations taken from the experimental data gives a good qualitative fit to the experimental time course. The experimental data (shown here only, independent of the experiments in Figure 2a) are the mean of 3 independent experiments testing predation of *K. pneumoniae* by *B. bacteriovorus* HD100 in Ca/HEPES, as described in the Methods.

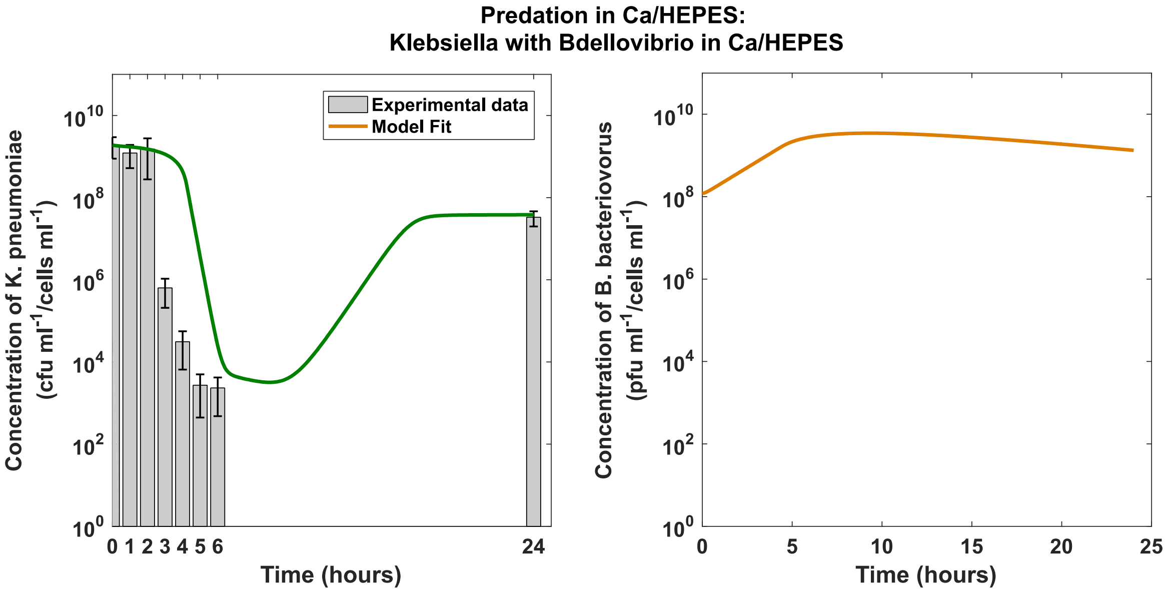

Supplementary Figure S8: Modelling predicts events beyond the scope of experimental data gathering for apparently inert experiments with slightly higher prey:predator MOI. The model simulation, extrapolated beyond the experimental time course, predicts a significant drop in prey concentration at 110 hours. The experimental data shown is the mean of three independent experiments testing the response of *K. pnuemoniae* to *B. bacteriovorus* in human serum.

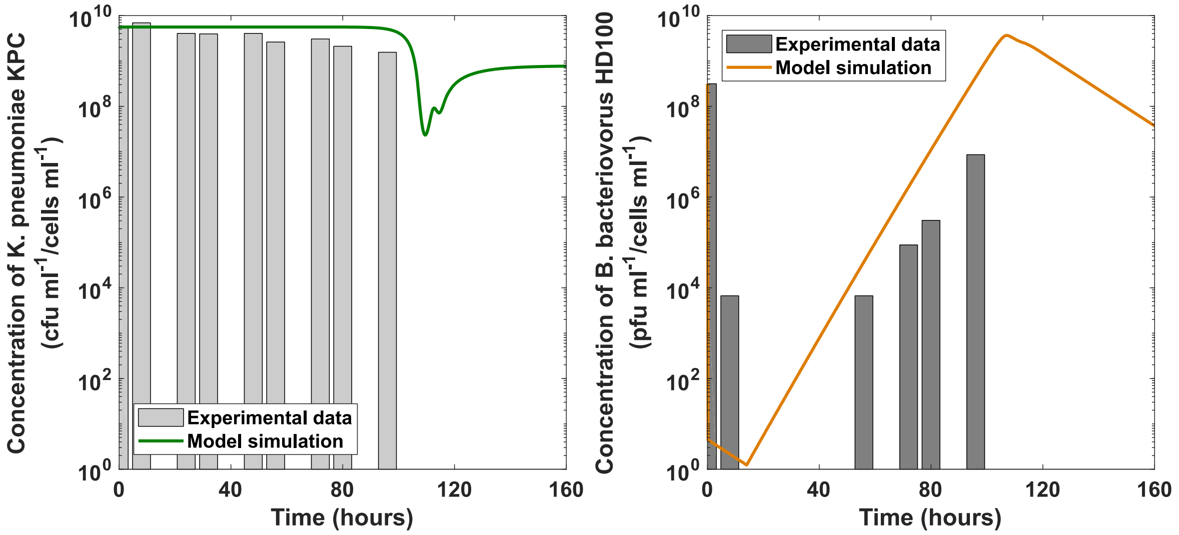

Supplementary Table S1: CH50 values for human serum incubated at 37°C. CH50 represents fold-dilution of human serum required to achieve 50% haemolysis of sensitized sheep erythrocytes. Serum was incubated alone (control serum), in the presence of *K. pneumoniae* (+ Kp), in the presence of *B. bacteriovorus* HD100 (+ Bd) or in the presence of both *K. pneumoniae* and *B. bacteriovorus* HD100 (+ Kp + Bd). ND= Not Determinable (haemolysis below 50%). Values are determined from the means of four technical replicates.

	**Time (h)**						
	**0**	**4**	**8**	**24**	**48**	**72**	**96**
**Control Serum**	16.72	9.54	4.88	ND	ND	ND	ND
**Serum + Kp**	12.98	ND	ND	ND	ND	ND	ND
**Serum + Bd**	13.62	ND	ND	ND	ND	ND	ND
**Serum + Kp + Bd**	9.72	ND	ND	ND	ND	ND	ND

Supplementary Table S2: Best fit parameter values used in the analysis of the mathematical model.

**Parameter**	**Best fit Parameters**			
	**Ca/HEPES buffer model (Fig 2B, C, D and 3)**	**HD100 alone in human serum (Fig 4C)**	***K. pneumoniae* alone in human serum**	**Predation in human serum (Fig 7 and 8)**
k_1^{early}	1.117×10^{-7}	-	-	5.890×10^{-14}
k_1^{late}		-	-	9.255×10^{-7}
k_2	1.453×10^{-3}	-	-	0.0035
k_3	6.960×10^{-5}	-	-	2.559×10^{-5}
k_4	0.830	-	-	0.8034
δ	-	-	-	10.82
V_{max}	-	-	1.153×10^{-4}	1.153×10^{-4}
V_{max}^{w}	0.0015	-	-	0.0018
E	-	-	4.932×10^{-5}	4.932×10^{-5}
E_w	2.605×10^{-6}	-	-	1.297×10^{-6}
s	-	-	9.695	9.695
s_w	251.4	-	-	367.3
d_P	6.673×10^{-8}	-	-	3.980×10^{-8}
d_{BD}	-	-	-	9.726×10^{-8}
d_I	-	0.1284	-	0.1284
d_{max}^{BD}	-	8.941×10^{-4}	-	8.941×10^{-4}
d_{min}^{BD}	-	3.547×10^{-4}	-	3.547×10^{-4}
h	-	1.977	-	1.977
a_{BD}	-	4.507×10^{-4}	-	4.507×10^{-4}
a_P		-	1.910×10^{-7}	1.910×10^{-7}
c_{BD}	-	1.152×10^{-7}	-	1.152×10^{-7}
c_P	-	-	2.594×10^{-6}	2.594×10^{-6}
f	1.227×10^{-13}	-	-	4.250×10^{-14}
n	-	-	0.4142	0.4142
ρ	3.85	-	-	3.85
I_0	-	98879.2	-	98879.2
R_0	-	-	258.2	258.2
ν	5.860×10^{-6}	-	-	5.860×10^{-6}
λ	2.646×10^{-5}	-	-	2.646×10^{-5}

Supplementary Table 3: List of experimental data used for parameter estimation and validation in the model

Parameters	Data sets used for parameter estimation	Data sets used for parameter validation
Buffer model parameters: $k_1; k_2; k_3; k_4; v_{max}^{w}; E_w; s_w; d_p; f; v; \lambda$	Predation in Ca/HEPES data (see Figure 2a) <br>3 independent biological repeats at 12 time point counts for prey controls, treated prey and predator (36 data points in total).	Predation in Ca/HEPES data (independent of data used for estimation, see Figure S2). Mean of 3 independent biological repeats with 8 time points counts for prey and initial t_0 count for predators (9 data points in total)
HD100 alone in human serum parameters: $d_I; d_{max}^{BD}; d_{min}^{BD}; h; a_{BD}; c_{BD}, I_0$	HD100 alone in serum data (see figure 4a). Mean of 2 independent biological repeats of 3 different experiments with different starting concentrations at 10 time points. (30 data points in total)	Parameters validated again data using in parameter estimation only.
K. pneumoniae alone in human serum parameters: $V_{max}; E; s; a_P; c_P; n, R_0$	*K. pneumoniae* alone in human serum data (see repeats 3 and 4 from Figure 5ai). 2 independent biological repeats at 9 time points (18 data points in total).	*K. pneumoniae* alone in human serum data (see repeats 1 and 2 from Figure 5ai) 2 independent biological repeats at 9 time points (18 data points in total). Results not shown.
Predation in human serum parameters: $k_1^{early}; k_1^{late}; k_2; k_3; k_4; \delta; V_{max}^{w}; E_w; s_w; d_p; d_{BD}; f$	Treated *K. pneumoniae* in human serum. Mean of 2 independent biological repeats from the same batch of serum at 10 time points (20 data points in total, see Figure 5, experiments 1 and 2).	Treated *K. pneumoniae* in human serum independent experiments with only k_1^{early} and δ estimated individually to take account of differences in experimental data.(6 sets of 20 data points, see Figure 5), mean of 3 independent experiments with late predation (see Figure S5). Independent parameter estimation on 3 independent data sets (see Figure S3).

1 Hobley, L., King, J. R. & Sockett, R. E. Bdellovibrio Predation in the Presence of Decoys: Three-Way Bacterial Interactions Revealed by Mathematical and Experimental Analyses. *Applied and Environmental Microbiology* **72**, 6757-6765, doi:10.1128/aem.00844-06 (2006).

2 Marchand, A. & Gabignon, O. [Theoretical model of the predator-prey interaction kinetics between "Bdellovibrio bacteriovorus" and "escherichia coli" (author's transl)]. *Ann Microbiol (Paris)* **132 B**, 321-336 (1981).

3 Varon, M. & Zeigler, B. P. Bacterial Predator-Prey Interaction at Low Prey Density. *Applied and Environmental Microbiology* **36**, 11-17 (1978).

4 Wilkinson, M. H. F. Predation in the Presence of Decoys: An Inhibitory Factor on Pathogen Control by Bacteriophages or Bdellovibrios in Dense and Diverse Ecosystems. *Journal of Theoretical Biology* **208**, 27-36, doi:http://dx.doi.org/10.1006/jtbi.2000.2197 (2001).

5 Ankomah, P. & Levin, B. R. Exploring the collaboration between antibiotics and the immune response in the treatment of acute, self-limiting infections. *Proceedings of the National Academy of Sciences* **111**, 8331-8338, doi:10.1073/pnas.1400352111 (2014).

6 Shemesh, Y. & Jurkevitch, E. Plastic phenotypic resistance to predation by Bdellovibrio and like organisms in bacterial prey. *Environmental Microbiology* **6**, 12-18, doi:10.1046/j.1462-2920.2003.00530.x (2004).

7 Lerner, T. R. *et al.* Specialized Peptidoglycan Hydrolases Sculpt the Intra-bacterial Niche of Predatory Bdellovibrio and Increase Population Fitness. *PLOS Pathogens* **8**, e1002524, doi:10.1371/journal.ppat.1002524 (2012).

8 Sockett, R. E. Predatory Lifestyle of Bdellovibrio bacteriovorus. *Annual Review of Microbiology* **63**, 523-539, doi:doi:10.1146/annurev.micro.091208.073346 (2009).

9 MATLAB, Optimization and Parallel computing toolbox Release 2015a (Natick, Massachusetts, United States. , 2016).

10 Dattner, I. *et al.* Modelling and parameter inference of predator–prey dynamics in heterogeneous environments using the direct integral approach. *Journal of The Royal Society Interface* **14**, doi:10.1098/rsif.2016.0525 (2017).

11 Esener, A. A., Roels, J. A. & Kossen, N. W. F. The influence of temperature on the maximum specific growth rate of Klebsiella pneumoniae. *Biotechnology and Bioengineering* **23**, 1401-1405, doi:10.1002/bit.260230620 (1981).

12 Rutgers, M., Teixeira De Mattos, M. J., Postma, P. W. & Van Dam, K. Establishment of the Steady State in Glucose-limited Chemostat Cultures of Klebsiella pneumoniae. *Microbiology* **133**, 445-451, doi:doi:10.1099/00221287-133-2-445 (1987).

13 Regué, M. *et al.* A Gene, uge, Is Essential for Klebsiella pneumoniae Virulence. *Infection and Immunity* **72**, 54-61, doi:10.1128/IAI.72.1.54-61.2004 (2004).

14 Kessel, M. & Shilo, M. Relationship of Bdellovibrio elongation and fission to host cell size. *Journal of Bacteriology* **128**, 477-480 (1976).
